# SUMO protease and proteasome recruitment at the nuclear periphery differently affect replication dynamics at arrested forks

**DOI:** 10.1093/nar/gkae526

**Published:** 2024-06-25

**Authors:** Kamila Schirmeisen, Karel Naiman, Karine Fréon, Laetitia Besse, Shrena Chakraborty, Anissia Ait Saada, Antony M Carr, Karol Kramarz, Sarah A E Lambert

**Affiliations:** Institut Curie, Université PSL, CNRS UMR3348, 91400 Orsay, France; Université Paris-Saclay, CNRS UMR3348, 91400 Orsay, France; INSERM U1068, CNRS UMR7258, Aix Marseille Univ U105, Institut Paoli-Calmettes, CRCM, Marseille, France; Genome Damage and Stability Centre, School of Life Sciences, University of Sussex, Falmer BN1 9RQ, UK; Institut Curie, Université PSL, CNRS UMR3348, 91400 Orsay, France; Université Paris-Saclay, CNRS UMR3348, 91400 Orsay, France; Institut Curie, Université PSL, CNRS UAR2016, Inserm US43, Université Paris-Saclay, Multimodal Imaging Center, 91400 Orsay, France; Institut Curie, Université PSL, CNRS UMR3348, 91400 Orsay, France; Université Paris-Saclay, CNRS UMR3348, 91400 Orsay, France; Institut Curie, Université PSL, CNRS UMR3348, 91400 Orsay, France; Université Paris-Saclay, CNRS UMR3348, 91400 Orsay, France; Genome Damage and Stability Centre, School of Life Sciences, University of Sussex, Falmer BN1 9RQ, UK; Academic Excellence Hub - Research Centre for DNA Repair and Replication, Faculty of Biological Sciences, University of Wroclaw, 50-328 Wroclaw, Poland; Institut Curie, Université PSL, CNRS UMR3348, 91400 Orsay, France; Université Paris-Saclay, CNRS UMR3348, 91400 Orsay, France; Equipe Labellisée Ligue Nationale Contre le cancer, France

## Abstract

Nuclear pore complexes (NPCs) have emerged as genome organizers, defining a particular nuclear compartment enriched for SUMO protease and proteasome activities, and act as docking sites for the repair of DNA damage. In fission yeast, the anchorage of perturbed replication forks to NPCs is an integral part of the recombination-dependent replication restart mechanism (RDR) that resumes DNA synthesis at terminally dysfunctional forks. By mapping DNA polymerase usage, we report that SUMO protease Ulp1-associated NPCs ensure efficient initiation of restarted DNA synthesis, whereas proteasome-associated NPCs sustain the progression of restarted DNA polymerase. In contrast to Ulp1-dependent events, this last function is not alleviated by preventing SUMO chain formation. By analyzing the role of the nuclear basket, the nucleoplasmic extension of the NPC, we reveal that the activities of Ulp1 and the proteasome cannot compensate for each other and affect the dynamics of RDR in distinct ways. Our work probes two distinct mechanisms by which the NPC environment ensures optimal RDR, both controlled by different NPC components.

## Graphical Abstract

**Graphical Abstract:**
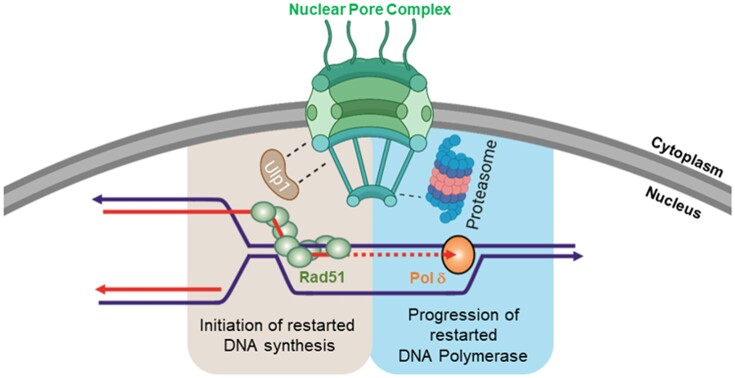

## Introduction

The eukaryotic genome is folded in 3D within a compartmentalized nucleus. Nuclear organization constitutes a critical layer of regulation of DNA-associated transactions and an important determinant of genome integrity (1). The stability of the genome is jeopardized during DNA replication; the progression of the replisome being recurrently threatened by a broad spectrum of obstacles that cause replication fork slowing, temporary fork stalling or terminal fork collapse (2). Such alterations of fork progression are a defining hallmark of replication stress. Failure to safeguard genome stability upon replication stress is a potent driving force behind the onset and progression of human diseases including cancer (3). While multiple replication fork repair pathways can be engaged at stressed forks to promote the completion of genome duplication, they result in variable outcomes for genome stability and thus must be carefully controlled and regulated. Our current knowledge of the regulatory functions played by nuclear organization in the usage of fork repair pathways remains in its infancy.

Among the fork repair pathways, homologous recombination (HR) is particularly active in protecting, repairing and restarting stressed forks, making HR an efficient tumor suppressor mechanism (4). The central factor of the HR machinery is the Rad51 recombinase that forms a nucleoprotein filament on single-stranded DNA (ssDNA) with the assistance of a loader, known as Rad52 in yeast models. In a non-recombinogenic mode, the Rad51 filament limits the degradation of ssDNA by various nucleases, thus ensuring the protection and integrity of stressed forks. In a recombinogenic mode, HR repairs broken forks with a single-ended double-strand break (DSB) by a mechanism called break-induced replication (BIR) and promotes replication resumption at DSB-free collapsed forks by a mechanism called recombination-dependent replication (RDR) (5). Both BIR and RDR are associated with non-canonical DNA synthesis, which is approximatively 100 times more mutagenic than canonical replication. Furthermore, during BIR and RDR, both DNA strands are synthesized by DNA polymerase delta (Pol δ) (6,7). These features allow experimental differentiation between DNA replicated by a repaired/restarted fork and DNA replicated by a canonical origin-born fork. Although stressed forks have the potential to relocate to the nuclear periphery (NP), little is known about the contribution of such changes in nuclear positioning in regulating the replicative functions of the HR machinery.

3D genome folding within the complex nuclear environment is a critical layer of DNA repair regulation. A striking example is the DNA damage response-dependent fate of DSBs that relocate to the NP or shift away from heterochromatic compartments to achieve error-free repair (8,9). This led to the concept that the membrane-less nuclear compartment exhibits distinct DNA repair capacities and that DNA repair machineries are spatially segregated. Nuclear pore complexes (NPCs) are macromolecular structures embedded in the nuclear envelope (NE) that act as nuclear scaffolds to regulate cellular processes via a wide range of mechanisms (10). The overall structure of NPCs is conserved among eukaryote kingdom, being composed of multiple copies of 30 different nucleoporins that associate in stable sub-complexes. The core NPC defines a central channel composed of transmembrane and channel nucleoporins. This core complex assembles with the outer and inner rings at the cytoplasmic and nuclear sides, respectively. A Y-shaped structure, located both at the cytoplasmic and nuclear side of NPCs, called in fission yeast the Nup107–Nup160 complex, is crucial for NPCs organization and proper segregation of chromosomes in eukaryotes (11–13). The final composition of individual NPCs is variable, depending on their position within the NE, suggesting that the NPC structure is dynamic. In particular, the nuclear basket, a nucleoplasmic extension of the core NPC, is the most dynamic part and NPCs localized in the nucleolar part of the NE are more frequently devoid of a nuclear basket (12). The primary functions of NPCs are the transport of macromolecules from the cytoplasm to the nucleus and mRNA export. NPCs also define a particular nuclear compartment enriched for the SUMO SENP protease and the proteasome and act as docking sites for DSBs and perturbed replication forks (8).

Stressed forks can relocate to the NP and, in some cases, anchor to NPCs (14). These include forks stalled by structure-forming DNA sequences, telomeric repeats, DNA-bound proteins and replication inhibitors (15–21). Although distinct scenarios arise depending on the source of replication stress and the model organism, the common emerging concept is that nuclear positioning of replication stress sites influences the usage of fork repair pathways. For example, in *Saccharomyces cerevisiae* (Sc), forks stalled within telomeric repeats associate with NPCs to restrict error-prone HR events and maintain telomere length (18). Forks stalled by CAG repeats, prone to form secondary DNA structure, also anchor to NPCs in a SUMO-dependent manner (16). In this instance, SUMOylated RPA on ssDNA at the stalled fork inhibits Rad51 loading, which is permitted only after NPC anchorage that subsequently favors error-free fork restart (17). Changes in nuclear positioning are far from being a yeast-specific phenomenon: upon DNA polymerase inhibition, stalled forks in human cells relocate to the NP to minimize chromosomal instability and ensure timely fork restart (20). Additionally, stressed forks at human telomeres relocate to NPCs to maintain telomere integrity (19).

We previously reported that, in the yeast *Schizosaccharomyces pombe* (Sp), dysfunctional forks relocate and anchor to NPCs in a SUMO-dependent manner, for the time necessary to achieve RDR (15). This change in nuclear positioning is critical to spatially segregate the subsequent steps of RDR. Dysfunctional forks are processed and remodeled in the nucleoplasm to load Rad51. SUMO chain, generated by the E3 SUMO ligase Pli1, then triggers relocation to NPCs. Relocation of dysfunctional forks to NPCs allows SUMO conjugates to be cleared by the SUMO deconjugating enzyme, Ulp1, which is sequestrated at the NP (22). Therefore, NPCs are an integral part of RDR regulation to promote HR-dependent DNA synthesis at dysfunctional forks. However, the dynamics underlying this process remain unexplored. In particular, the contribution of NPCs to non-canonical Pol δ/Pol δ DNA synthesis, a hallmark of HR-restarted forks, has not been addressed. Here, by mapping DNA polymerase usage during HR-mediated fork restart, we reveal that the SUMO protease, Ulp1, and the proteasome differentially affect the dynamics of HR-dependent fork restart by ensuring efficient resumption of DNA synthesis and by sustaining the dynamic progression of the restarted fork, respectively. Moreover, by studying the role of the nuclear basket in RDR, we show that Ulp1 and the proteasome do not compensate for each other. In particular, the defect in RDR caused by defective Ulp1-associated NPCs, but not defective proteasome-enriched NPCs, is alleviated by preventing SUMO chain formation. Our study uncovers mechanisms by which the NPC compartment acts as a critical environment for optimal HR-dependent fork restart.

## Materials and methods

### Standard yeast genetics and biological resources

Yeast strains and primers used in this work are listed in Supplementary Table S1 and S2, respectively. Gene deletion and tagging were performed by classical genetic techniques. To assess the sensitivity of chosen mutants to genotoxic agents, mid log-phase cells were serially diluted and spotted onto yeast extract agar plates containing hydroxyurea (HU), methyl methanesulfonate (MMS), camptothecin (CPT) or bleomycin (bleo). Strains carrying the *RTS1* replication fork block sequence were grown in minimal medium EMMg (with glutamate as a nitrogen source) with addition of appropriate supplements and 60 μM thiamine (barrier inactive, OFF). The induction of replication fork block was obtained by washing away the thiamine and further incubation in a fresh medium for 24 hours (barrier active, ON).

### Live cell imaging

For snapshot microscopy, cells were grown in filtered EMMg with or without 60 μM thiamine for 24 h to exponential phase (RFB OFF and RFB ON), then centrifuged and resuspended in 500 μl of fresh EMMg. 1 μl from the resulting solution was dropped onto Thermo Scientific slide (ER-201B-CE24) covered with a thin layer of 1.4% agarose in filtered EMMg (15). 21 z-stack pictures (each z step of 200 nm) were captured using a Nipkow Spinning Disk confocal system (Yokogawa CSU-X1-A1) mounted on a Nikon Eclipse Ti E inverted microscope, equipped with a 100× Apochromat TIRF oil-immersion objective (NA: 1.49) and captured on sCMOS Prime 95B camera (Photometrics) operated through MetaMorph^®^ software (Molecular Devices). GFP and mCherry proteins were excited with a 488 nm (Stradus® - Vortran Laser Technology, 150mW) and a 561 nm (Jive™-Cobolt, 100 mW) lasers, respectively. A quad band dichroic mirror (405/488/568/647 nm, Semrock) was used in combination with single band-pass filters of 525/50 or 630/75 for the detection of GFP and mCherry, respectively. Fluorescence and bright-field 3D images were taken at every 0.3μm over a total of 4.5μm thickness. Exposure time for the GFP channel was 500 ms and for the mCherry channel was 1000 ms. During the imaging, the microscope was set up at 25°C. For the experiment on Ulp1 and Cut11, the Gataca Live SR module (Gataca Systems), implemented on the Spinning Disk confocal system, was used to generate super-resolution images with lateral image resolution improvement (around 120 nm).

### Image analysis

Images were mounted and analyzed with Fiji software (23). First, the 3D Z series are converted into 2D projection based on maximum intensity values. The quantification of Ulp1 and Cut11 was performed using a homemade macro. The user draws manually all nuclear rings on the merge images as a first step. Then automatically, three types of regions were created from the manual annotation:

- the nucleus was obtained by enlarging the manual annotation to 3 pixels.
- the nucleoplasm was obtained by shrinking the nucleus to 8 pixels.
- the nuclear periphery has been extracted from the previous two regions by selecting only those pixels that are not common.

Several measurements were exported for all regions, such as perimeter of nuclei in pixels, area in pixels², the fluorescence density of a protein (annotated as ‘Mean fluorescence intensity’ in Fiji: this value represents the Raw Integrated Density measured in the selection and normalized by the area of the same selection) and the total fluorescence intensity of the protein (annotated as ‘RawIntDen’(Raw Integrated Density) in Fiji: this value represents the sum of all pixels intensities in the selection). To assess the co-localization of Ulp1 and Cut11 proteins, the JACoP plugin (24) was used to study the correlation between the intensities of these two proteins in different NPC mutant strains. Pearson and Manders’ coefficients were calculated for each nucleus obtained. Before running the analysis, pre-processing was applied (background subtraction using the rolling ball algorithm with a radius of 20 pixels and a Gaussian filter (sigma 1)) to reduce image noise and facilitate detection of the Ulp1 and Cut11 proteins needed to calculate Manders’ coefficients. The ‘Default’ thresholding method was used for the detection of Ulp1-GFP and Cut11-mCherry positive signals.

## 2DGE analysis of replication intermediates

Exponential cells (2.5 × 10^9^) were treated with 0.1% sodium azide and subsequently mixed with frozen EDTA (of final concentration at 80 mM). Genomic DNA was crosslinked with trimethyl psoralen (0.01 mg/mL, TMP, Sigma, T6137) which was added to cell suspensions and incubated for 5 min in the dark. Next, cells were irradiated with UV-A (365 nm) for 90 s at a constant flow of 50 mW/cm^2^. Subsequently, cell lysis was performed by adding lysing enzymes (Sigma, L1412) at a concentration of 0.625 mg/ml and zymolyase 100 T (Amsbio, 120493-1) at 0.5 mg/ml. Obtained spheroplasts were next embedded into 1% low melting agarose (InCert Agarose 50123, Lonza) plugs and incubated overnight at 55°C in a digestion buffer with 1 mg/ml of proteinase K (Euromedex EU0090). Plugs were then washed with TE buffer (50 mM Tris, 10 mM EDTA) and stored at 4°C. Digestion of DNA was performed using 30 units of restriction enzyme *Ase*I (NEB, R0526M) per plug. Samples were then treated with RNase (Roche, 11119915001) and beta-agarase (NEB, M0392L). Melted plugs were equilibrated to 0.3 M NaCl. Replication intermediates were purified using BND cellulose (Sigma, B6385) poured into columns (Biorad, 731-1550) (25). RIs were enriched in the presence of 1M NaCl 1.8% caffeine (Sigma, C-8960), precipitated with glycogen (Roche, 1090139001) and migrated in 0.35% agarose gel (1× TBE) for the first dimension. The second dimension was cast in 0.9% agarose (1× TBE) supplemented with 0.5 μg/ml of EtBr. Next, DNA was transferred to a nylon membrane (Perkin-Elmer, NEF988001PK) in 10× SSC. Finally, membranes were incubated with ^32^P-radiolabeled *ura4* probe (TaKaRa *Bca*BEST^TM^ Labeling Kit, #6046 and alpha-^32^P dCTP, Perkin-Elmer, BLU013Z250UC) in Ultra-Hyb buffer (Invitrogen, AM8669) at 42°C. The signal of replication intermediates was collected in phosphor-imager software (Typhoon-trio) and quantified by densitometric analysis with ImageQuantTL software (GE healthcare). The ‘tail signal’ was normalized to the overall signal corresponding to arrested forks.

### Replication slippage assay

The frequency of *ura4 +* revertants arising from the *ura4-sd20* allele was determined as follows. 5-FOA (EUROMEDEX, 1555) resistant colonies were grown on plates containing uracil with or without thiamine for 2 days at 30°C and subsequently inoculated into EMMg supplemented with uracil for 24 h. Then cultures were diluted and plated on EMMg complete (for cell survival) and on EMMg without uracil, both supplemented with 60 μM thiamine. After 5–7 days of incubation at 30°C colonies were counted to determine the frequency of *ura4+* reversion.

### Flow cytometry

Flow cytometry analysis of DNA content was performed as follows (26): cells were fixed in 70% ethanol and washed with 50 mM sodium citrate, digested with RNAse A (Sigma, R5503) for 2 h, stained with 1μM Sytox Green nucleic acid stain (Invitrogen, S7020) and subjected to flow cytometry using FACSCANTO II (BD Biosciences).

### Whole protein extract analysis

Aliquots of 1 × 10^8^ cells were collected and disrupted by bead beating in 1 ml of 20% TCA (Sigma, T9159). Pellets of denatured proteins were washed with 1 M Tris pH 8 and resuspended in 2× Laemmli buffer (62.5 mM Tris pH 6.8, 20% glycerol, 2% SDS, 5% β-mercaptoethanol with bromophenol blue). Samples were boiled before being subjected to SDS-PAGE on Mini-PROTEAN TGX Precast Gel 4-15% (Biorad, 4561086). Western blot using either anti-GFP (Roche, 11814460001), anti-HA (Santa Cruz Biotechnology, sc-57592), anti-TIR1 (MBL, PD048), anti-PCNA (Santa Cruz, sc-56) or anti-tubulin (Abcam, Ab6160) antibodies was subsequently performed. For the analysis of cellular patterns of global SUMOylation, whole protein extraction was performed as follows: aliquots of 2 × 10^8^ cells were collected and resuspended in 400 μl of water. The cell suspensions were mixed with 350 μl of freshly prepared lysis buffer (2M NaOH, 7% β-mercaptoethanol) and 350 μl of 50% TCA (Sigma, T9159). After centrifugation, pellets were further washed with 1 M Tris pH 8 and resuspended in 2× Laemmli buffer (62.5 mM Tris pH 6.8, 20% glycerol, 2% SDS, 5% β-mercaptoethanol with bromophenol blue). Samples were boiled before being subjected to SDS-PAGE on Mini-PROTEAN TGX Precast Gel 4–15% (Biorad, 4561086). Western blot using anti-SUMO antibody (non-commercial, produced in rabbit by Agro-Bio) was subsequently performed.

### Pulse field gel electrophoresis

Yeast cultures were grown to logarithmic phase in rich YES medium to a concentration of 5 × 10^6^/ml, synchronized in 20 mM HU for 4 h, and subsequently released to fresh YES medium. At each time point, 20 ml of cell culture was harvested, washed with cold 50 mM EDTA pH 8 and digested with lyticase (Sigma, L4025) in CSE buffer (20 mM citrate/phosphate pH 5.6, 1.2 M sorbitol, 40 mM EDTA pH 8). Next cells were embedded into 1% UltraPure™ Agarose (Invitrogen, 16500) and distributed into 5 identical agarose plugs for each time point. Plugs were then digested with Lysis Buffer 1, LB1 (50 mM Tris-HCl pH 7.5, 250 mM EDTA pH 8, 1% SDS) for 1.5 h at 55°C and transferred to Lysis Buffer 2, LB2 (1% *N*-lauryl sarcosine, 0.5 M EDTA pH 9.5, 0.5 mg/ml proteinase K) o/n at 55°C. The next day, LB2 was exchanged to for fresh LB2 and digestion was continued o/n at 55°C. After this, plugs were kept at 4°C. To visualize intact chromosomes, one set of plugs was run on a Biorad CHEF-DR-III pulse field gel electrophoresis (PFGE) system for 60 h at 2.0 V/cm, angle 120°, 14°C, 1800 s single switch time, pump speed 70 in 1× TAE buffer. Separated chromosomes were stained in ethidium bromide (10 μg/ml) for 30 min, washed briefly in 1× TAE and visualized with a UV trans-illuminator.

### Npp106-GFP chromatin immuno-precipitation

Chromatin immunoprecipitation against Npp106-GFP was done as described earlier (15) with the following modifications: 200 ml of logarithmic yeast culture (OD_600_∼ 1) for each strain and condition (*RTS1-*RFB OFF and ON) was divided into two 100ml aliquots and double-cross-linked first with 10 mM dimethyl adipimidate (DMA, Thermo scientific, 20660) and then with 1% formaldehyde (Sigma, F-8775). Next, cells were frozen in liquid nitrogen. Cell lysis was performed by bead beating in 400 μl of lysis buffer (50 mM HEPES pH7.5, 140 mM NaCl, 1% Triton X100, 0.1% Nadeoxycholate, 1 mM EDTA plus 1 mM PMSF and protease inhibitors, Sigma-Aldrich, P8215). Sonication of chromatin was done in a Diagenode Bioruptor Pico in Easy Mode, 10 cycles, 30s ON and 30s OFF at 4°C. Sonicated chromatin fractions were pooled for each condition. 5 μl of sonicated chromatin was preserved as the input fraction. Immunoprecipitation was carried out over night as follows: 300 μl of sonicated chromatin extract was incubated with anti-GFP antibody (Invitrogen, A11122) at 1:150 concentration and another 300 μl was incubated with Normal Rabbit IgG antibody (Cell Signaling Technology, #2729S) at dilution 1:75. The next morning, protein G Dynabeads (Invitrogen, 10003D) were added for 1 h. Immunoprecipitated proteins and inputs were decrosslinked for 2 h at 65°C. Inputs and the DNA associated with Npp106-GFP or control Rabbit antibody were purified using Qiaquick PCR purification kit (QIAGEN, 28104) and eluted in 400 μl of water for molecular biology (Sigma-Aldrich, 95284). Quantitative PCR was performed using SsoAdvanced Universal SYBR® Green Supermix (#1725274, Biorad). Primers used for qPCR are listed in Supplementary Table S2. The relative amounts of DNA, starting quantities based on standard curves for each pair of primers, were obtained using Biorad CFX Maestro 1.1.

### Pu-Seq

The published protocol (27) was used with minor modifications: size selection was performed using a Blue Pippin (Sage Science). We used rnh201-RED instead of rnh201::kan strain (28). Sequence files were aligned with Bowtie2 and alignment data converted to counts with custom Perl script (27). Analysis of polymerase usage was performed with custom R script (27). Sequence data is available under GEO dataset GSE247371.

**Figure 1.**
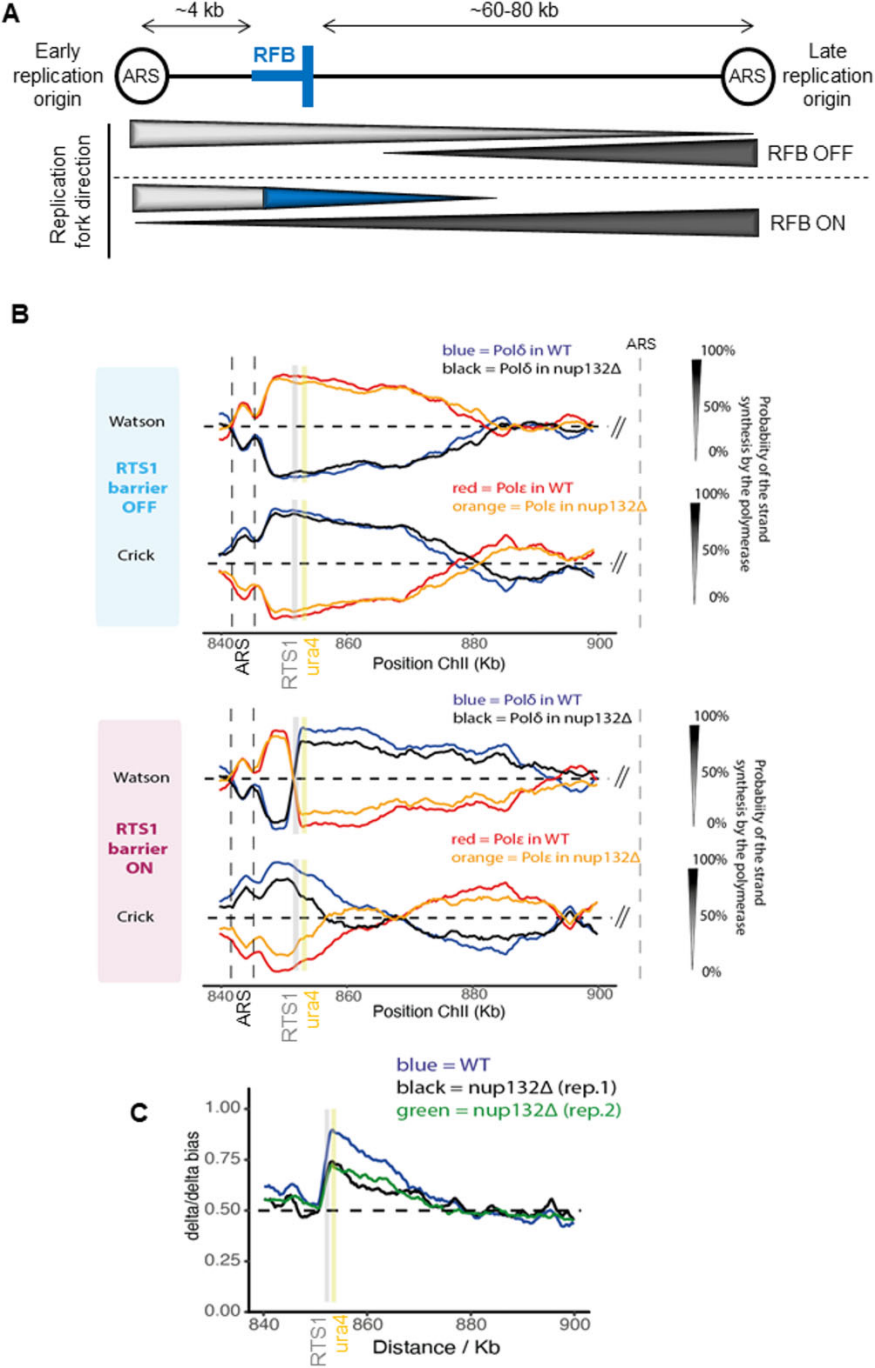
Ulp1-associated NPC promotes the dynamics of recombination-mediated fork restart. (**A**) Schematic of the *RTS1*-RFB locus on chromosome II. The position of the *RTS1*-RFB is indicated as thick blue bars. The directional RFB blocks the progression of right-moving forks that initiate from the left autonomously replicating sequence (ARS). The direction of unperturbed (RFB OFF) and perturbed replication (RFB ON) forks is indicated by the thickness of the arrows underneath. Light and dark gray bars indicate the progression of canonical rightward and leftward-moving forks, respectively. The blue bar indicates the progression of restarted replication forks mediated by homologous recombination. (**B**) Pu-Seq traces of the ChrII locus in *RTS1*-RFB OFF (top panel) and ON (bottom panel) conditions in WT and *nup132Δ* strains. The usage of Pol delta (in blue and black for WT and *nup132Δ* cells, respectively) are shown on the Watson and Crick strands. The usage of Pol epsilon (in red and orange for WT and *nup132Δ* cells, respectively) are shown on the Watson and Crick strands. Note the switch from Pol epsilon to Pol delta on the Watson strand at the RFB site (gray bar), which is indicative of a change in polymerase usage on the leading strand in RFB ON condition. The genomic location of the ARS, the *RTS1*-RFB and the *ura4* marker are indicated by dashed lines, a gray line and a yellow line, respectively. (**C**) Graph of Pol delta/delta bias over both strands (Watson and Crick) around the RFB site in WT and two independent replicates of *nup132Δ* strains. The gray and yellow bars indicate the position of the *RTS*1-RFB and of the *ura4* marker, respectively.

The percentage of forks that restart at the barrier is estimated from the delta/delta bias plots (Figures 1C and 6C). Immediately downstream of the site of the barrier the level of increase in delta/delta bias is indicative of percentage of restarted forks (1 = 100% restarted; 0.5 = 0% restarted). The relative progression of the restarted forks is estimated from the relative change in slope of the delta/delta bias score as the forks progress from the barrier towards the right. This reflects the termination of the restarted forks as they meet canonical forks progressing leftwards: slow restarted forks will terminate closer to the barrier.

## Statistical analysis

Quantitative densitometric analysis of Southern blots after 2DGE was carried out using ImageQuant software. The ‘tail signal’ of resected forks was normalized to the overall signal corresponding to arrested forks.

Quantification of PFGE was performed using ImageJ and presented as % of migrating chromosomes relative to asynchronous profile. Cell imaging was performed using METAMORPH software and processed and analyzed using ImageJ software (23). The explanation and definition of values and error bars are mentioned within the figure legends. In most experiments, the number of samples is >3 and obtained from independent experiments to ensure biological reproducibility. For all experiments based on the analysis of cell imaging, the number of nuclei analyzed is mentioned in the figure legends. Statistical analysis was carried out using Mann–Whitney *U* test, Student's *t*-test and Fischer's exact test.

## Results

To investigate the contribution of the NP to the dynamics of HR-mediated fork restart, we exploited the *RTS1* replication fork barrier (RFB) that promotes the polar arrest of a single replisome at a specific genomic location (Figure 1A) (25). The activity of the RFB is fully dependent on the Rtf1 protein that binds to the *RTS1* sequence. The expression of Rtf1 can be artificially regulated by the *nmt41* promoter, which allows Rtf1 repression in thiamine-containing media (RFB OFF condition) and its expression upon thiamine removal (RFB ON condition). Alternatively, the *rtf1* gene can be deleted and the results compared with an *rtf1*+ strain. Forks arrested at the RFB become fully dysfunctional and undergo controlled degradation of the nascent strand by the end-resection machinery to generate ssDNA gap of ∼1 kb in length (29,30). RPA, Rad52 and Rad51 are loaded onto these ssDNA gaps, ensuring fork protection until the arrested fork is either fused with a converging fork or actively restarted by RDR, which occurs approximately 20 minutes after the arrest (6,28,31,32). The restarted fork is associated with a non-canonical, mutagenic DNA synthesis in which both strands are synthesized by Pol δ, making it insensitive to the RFB (28,31,33,34).

### Ulp1-associated NPCs ensure the efficient priming of recombination-mediated DNA synthesis

We previously reported that the nucleoporin Nup132, part of the Y complex of NPCs core, promotes RDR in a post-anchoring manner and acts downstream of Rad51 loading (15). The RDR defect observed in *nup132* null cells is caused by the delocalization of the Ulp1 SUMO protease from the NP since the artificial tethering of Ulp1 to the RFB, anchored to NPCs, restored RDR efficiency. Thus, Ulp1-associated NPCs prime HR-dependent DNA synthesis to ensure efficient RDR, but the dynamics of this process is unknown. To address this, we employed the polymerase usage sequencing (Pu-Seq) approach that allows the genome-wide mapping of the usage of Pol δ and polymerase epsilon (Pol ε) during DNA replication (35). Pu-Seq makes use of a pair of yeast strains mutated in either Pol δ or Pol ε that incorporate higher levels of ribonucleotides during DNA synthesis. The mapping of ribonucleotides in a strand-specific manner in strains mutated either for Pol δ or Pol ε allows the genome-wide tracking of polymerase usage. Combined with the *RTS1*-RFB, the Pu-Seq method allows monitoring the usage frequency of each polymerase separately on both the Watson and Crick strand when the RFB is either inactive (RFB OFF, in an *rtf1Δ* genetic background) or constitutively active (RFB ON, Rtf1 expressed from the *adh1* promoter to maximize fork arrest efficiency) (28).

At an inactive barrier site (RFB OFF), replication is canonical (leading strand synthesized by Pol ε and lagging strand synthesized by Pol δ) and proceeds from left to right in the majority of cells, initiating from an early replication origin (Figure 1A-B, top panel). This division of labor between Pol δ and ε changed sharply in an RFB ON strain: at the barrier site, Pol ε in the leading strand is switched to Pol δ during the restart of the blocked fork (Figure 1B, bottom panel). This sharp transition characterizes the efficiency of the restart itself. It means that this creates a bias towards Pol δ when considering both strands (Watson and Crick) downstream of the *RTS1*-RFB site due to the restart. The Pol δ/δ bias reflects the time needed for the restart as well as the progression of the restarted fork relative to the canonical convergent fork coming from a late replication origin (28). Based on the Pol δ/δ bias (Figure 1C), we estimated that, when compared to WT (*nup132*+) cells, only 60% of the expected number of forks were arrested and restarted in *nup132Δ* cells, while the remaining 40% were either not arrested or were arrested and did not restart before being rescued by an incoming leftward moving canonical fork. The increase in Pol ε usage on the Crick strand for ∼10 Kb downstream of the *RTS1* barrier is indicative of this latter scenario (Figure 1B). Remarkably, this fork-restart defect is consistent with our previous estimation using a proxy-restart assay that exploits the mutagenic nature of restarted DNA synthesis to provide a genetic readout of RDR efficiency. Using this proxy-restart assay, we previously reported a nearly two-fold reduction in RDR efficiency in *nup132Δ* cells compared to WT (15). Finally, the relative change in slope of the Pol δ/δ bias reduction over distance was similar between the two replicates from *nup132Δ* cells and the WT strain, indicating that the forks that succeeded to restart progress with similar speed (Figure 1C).

### The nuclear basket promotes RDR in a pre- and post-anchoring manner

We next investigated the role of the nuclear basket in dealing with replication stress. The *S. pombe* nuclear basket is composed of 4 non-essential nucleoporins: Nup60 (ScNup60), Nup61 (ScNup2, HsNup50), Nup124 (ScNup1, HsNup153) and Alm1 (ScMlp1/2, HsTPR) (12, 13). A fifth component is the essential nucleoporin Nup211, a second orthologue of ScMlp1/2 and HsTPR. Some of these components are known to contribute to resistance to DNA damage (36,37). We confirmed that *alm1Δ* cells were highly sensitive to a wide range of replication-blocking agents and bleomycin-induced DSBs, whereas *nup60Δ* and *nup61Δ* cells exhibited mild sensitivity only to hydroxyurea (HU), a replication inhibitor that depletes dNTP pool (Supplementary Figure S1A).

To establish if this HU sensitivity correlates with a defect in resuming replication following HU treatment, we arrested cells for 4 h in 20mM HU and then followed DNA content by flow cytometry upon release into HU-free media. Among nuclear basket mutants, only *nup61Δ* cells displayed a defect in the recovery from HU-stalled forks, a defect similar to the one previously reported for *nup132Δ* cells (15) (Supplementary Figure S1B): the WT strain reached a G2 DNA content 45 minutes after release, whereas both *nup132Δ* and *nup61Δ* cells exhibited an additional 15 minutes delay. This observation is supported by the analysis of chromosomes by Pulse Field Gel Electrophoresis (PFGE). HU treatment prevented chromosomes from migrating into the gel because of the accumulation of replication intermediates (Supplementary Figure S1C). WT chromosomes migrated into the gel with twice intensity of an asynchronous culture 90 minutes after release, indicating a complete recovery from HU-stalling forks and genome duplication of the WT genome (Supplementary Figure S1D). Consistent with the flow cytometry data, only chromosomes from *nup132Δ* and *nup61Δ* cells showed a clear delay in their ability to migrate into the gel following release from HU, confirming a role for Nup61 in promoting DNA replication upon transient fork stalling by HU.

**Figure 2.**
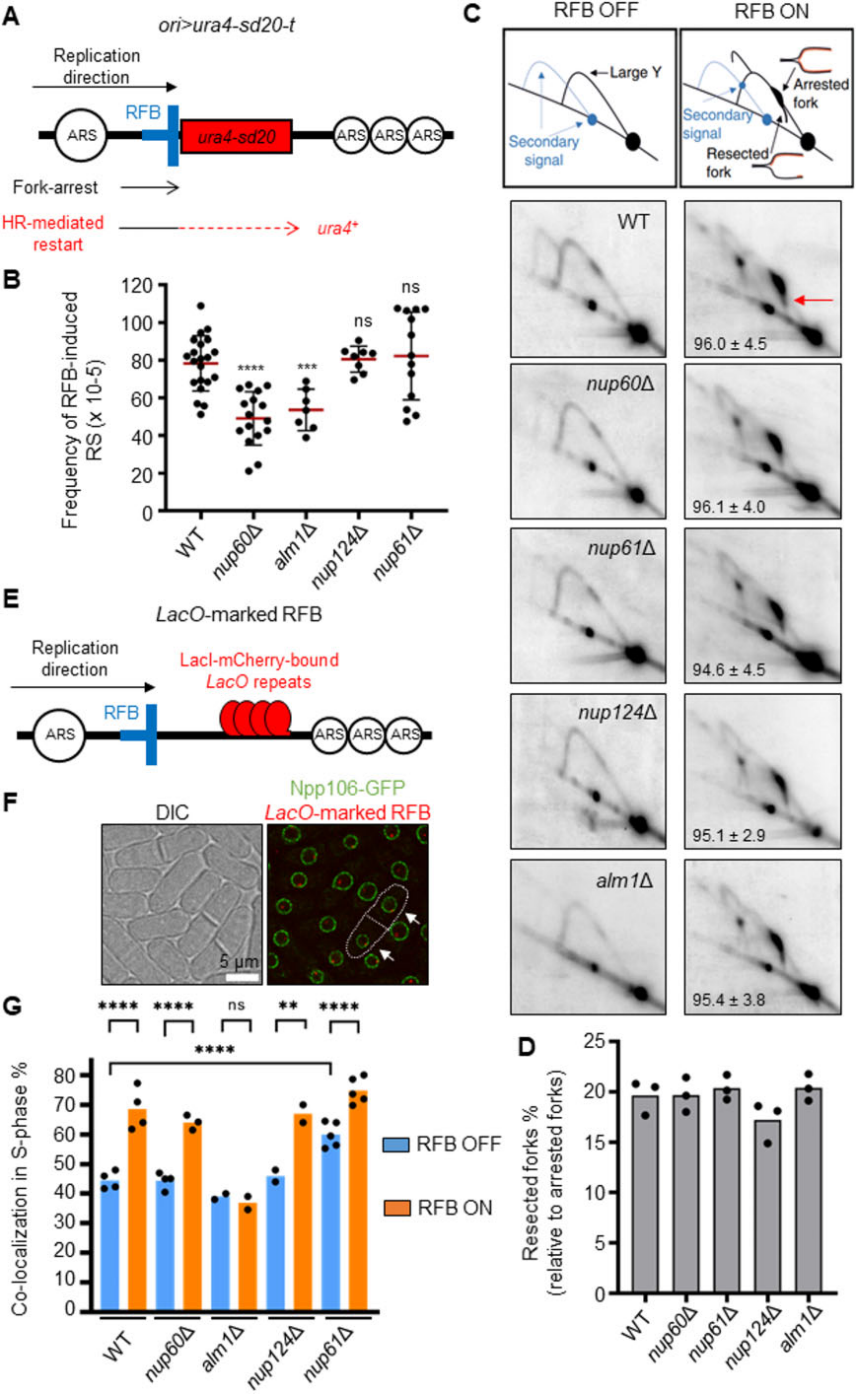
The nuclear basket promotes recombination-dependent replication in a pre- and post-anchoring manner. (**A**) Diagram of the *ori > ura4-sd20-t* construct on chromosome III (ori: replication origin, >: *RTS1*-RFB orientation that block right-moving forks, t: telomere). The non-functional *ura4-sd20* allele (red box), containing a 20 nt duplication flanked by micro-homology, is located downstream of the RFB (blue bar). During HR-mediated fork restart, the *ura4-sd20* allele is replicated by an HR-associated DNA synthesis that is liable to replication slippage (RS) resulting in the deletion of the duplication and the restoration of a functional *ura4^+^* gene (34). ARS: autonomously replicating sequence. (**B**) Frequency of RFB-induced RS in indicated strains. Each dot represents one sample from independent biological replicate. Red bars indicate mean values ± standard deviation (SD). *P* value was calculated by two-sided *t*-test (**** *P* ≤ 0.0001; *** *P* ≤ 0.001; ns: non-significant). (**C**) Top panel: scheme of replication intermediates (RI) analyzed by neutral-neutral 2DGE of the *AseI* restriction fragment in RFB OFF and ON conditions. Partial restriction digestion caused by psoralen-crosslinks results in a secondary arc indicated on scheme by blue dashed lines. Bottom panels: representative RI analysis in indicated strains and conditions. The *ura4* gene was used as a probe. Numbers indicate the % of forks blocked by the RFB ± SD. The red arrow indicates the tail signal resulting from resected forks. (**D**) Quantification of resected forks in indicated strains. Dots represent values obtained from independent biological experiments. No statistical difference was detected between the samples using the two-sided *t*-test. (**E**) Diagram of the *LacO*-marked RFB. *LacO* arrays bound by mCherry-LacI (red ellipses) are integrated ∼7 kb away from the *RTS1*-RFB (blue bar). (**F**) Example of fluorescence (right panel) and bright-field images (left panel, DIC) cells expressing the endogenous Npp106-GFP fusion protein and harboring the *LacO*-marked RFB. Mono-nucleated cells and septated bi-nucleated cells correspond to G2 and S-phase cells, respectively. White arrows indicate co-localization events in S-phase cells. Scale bar: 5 μm. (**G**) Quantification of co-localization events, shown in f, in S-phase cells in indicated conditions and strains. Dots represent values obtained from independent biological experiments. At least 100 nuclei were analyzed for each strain and condition. Fisher's exact test was used for group comparison to determine the *P* value (****P ≤ 0.0001; ** *P* ≤ 0.01; ns: non-significant).

To establish the role of the nuclear basket in promoting replication resumption at the RFB, we first measured replication slippage (RS) downstream of *RTS1*, a proxy measure of non-canonical replication resulting from RDR (34) (Figure 2A). The absence of Nup60 and Alm1, but not Nup124 or Nup61, led to a ∼2-fold reduction in the frequency of RFB-induced RS, indicating a reduced RDR efficiency (Figure 2B). The analysis of replication intermediates by bi-dimensional gel electrophoresis (2DGE) showed that fork arrest and the formation of large ssDNA gaps (>100 bp) at the RFB (which are visualized as a specific ‘tail’ DNA structure emanating from the fork arrest signal and descending toward the linear arc; see red arrow on Figure 2C) (32) were unaltered in all four non-essential nucleoporin mutants (Figure 2C, D). This indicates that the controlled degradation of the nascent strand and Rad51-dependent fork protection are unaffected. Thus, the RDR defect observed in *nup60Δ* and *alm1Δ* is not related to defect in the early steps of RDR, from ssDNA gap formation to Rad51 loading.

We next investigated the ability of the RFB to relocate to the NP. We employed a strain harboring a *LacO*-marked RFB expressing LacI-mCherry (Figure 2E) and an endogenously GFP-tagged Npp106, a NPC component, to mark the NP (Figure 2F). We counted co-localization events between the NP and the *LacO*-marked RFB, visualized by a LacI-mCherry focus (see white arrows on Figure 2F), as previously reported (15). When the RFB was inactive (RFB OFF), LacI foci co-localized with the NP in ∼45% of S-phase cells (Figure 2G). Upon activation of the RFB (RFB ON), the *LacO*-marked RFB was more often (∼70%) localized at the NP in WT cells (15). This shift of the active RFB to the NP was observed in all nuclear basket mutants, except *alm1Δ* (Figure 2G). The *nup61Δ* cells exhibited a slight increase in the frequency of co-localization events in RFB OFF condition but reached a similar enrichment at the NP to WT cells in RFB ON condition. Thus, Alm1 and Nup60 promote RDR in a pre- and post-anchoring manner, respectively.

### The nuclear basket promotes the sequestration of Ulp1 at the nuclear periphery

**Figure 3.**
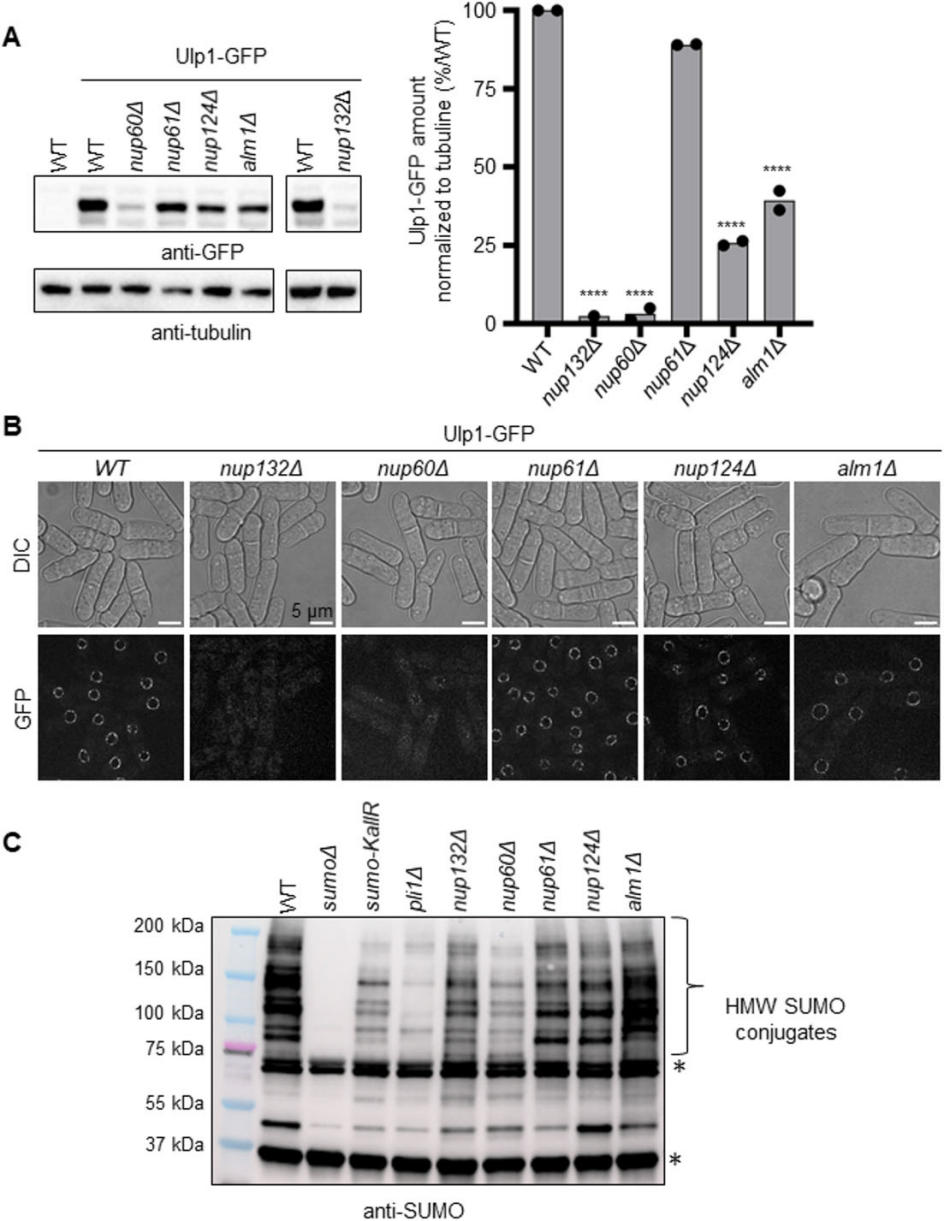
The nuclear basket regulates the expression of the SUMO SENP protease Ulp1. (**A**) Left panel: expression of Ulp1-GFP in indicated strains. An untagged WT strain was included as control for antibody specificity. Tubulin was used as a loading control. Right panel: quantification. The normalized amount of Ulp1 was calculated by dividing the GFP signal by tubulin signal. The normalized amount of Ulp1-GFP in the mutants is indicated as a percentage of the WT. Dots represent values obtained from independent biological experiments. *P* value was calculated by two-sided *t-*test (**** *P* ≤ 0.0001). (**B**) Example of fluorescence (bottom panel) and bright-field images (top panel, DIC) of cells expressing the endogenous Ulp1-GFP fusion protein in indicated strains. Scale bar 5 μm. (**C**) Expression of SUMO conjugates in indicated strains. A strain deleted for *pmt3* gene that encodes the SUMO particle (*sumoΔ*) was added as control for antibody specificity. * indicates unspecific signal.

In budding yeast, several components of the nuclear basket are critical for peripheral Ulp1 localization, including ScNup60 and the synergistic action of ScMlp1 and ScMlp2 (38,39). We thus investigated the expression and the nuclear sub-localization of Ulp1 upon loss of nuclear basket functionality. Ulp1 was C-terminally tagged with GFP and Ulp1-GFP functionality was established using resistance to genotoxic stress (Supplementary Figure S2A). We observed that, in *nup60Δ* and *nup132Δ* mutants, Ulp1-GFP levels were largely abrogated whereas a ∼75% and ∼60% reduction was observed in *nup124Δ* and *alm1Δ* backgrounds, respectively (Figure 3A). Cell microscopy analysis showed that Ulp1-GFP was no longer sequestrated at the NP in *nup60Δ* and *nup132Δ* mutants (Figure 3B). Treating cells with bortezomib, a proteasome inhibitor (40), partly restored Ulp1-GFP protein level in *nup132Δ* and *nup60Δ* cells, similar to previous findings in budding yeast (38). However, the sequestration of Ulp1-GFP at the NP was far from being fully restored (Supplementary Figure S2B, C).

In *S. pombe*, Ulp1 delocalization leads to the degradation of the SUMO chain-modified Pli1, an E3 SUMO ligase, resulting in a global decrease of SUMO conjugates (41). Consistent with Ulp1 expression being severely lowered and delocalized from the NP in *nup132Δ* and *nup60Δ* (Figure 3A and B), we observed a global reduction in the accumulation of SUMO conjugates when compared to WT (Figure 3C). The pattern of SUMO conjugates in *nup132Δ* and *nup60Δ* backgrounds was similar to that observed in a strain expressing SUMO-KallR, in which all internal lysine are mutated to arginine to prevent SUMO chain formation (15). The accumulation of SUMO conjugates was more adversely affected by the absence of Pli1 than in *nup132Δ* and *nup60Δ* cells, suggesting that Pli1 maintains some activity in these genetic backgrounds, as reported for *nup132Δ* cells (15). Despite the reduced Ulp1 expression in *nup124Δ* and *alm1Δ* cells, the pattern of SUMO conjugates was less affected, suggesting that the remaining Ulp1 is sequestered properly at the NP in these genetic backgrounds (Figure 3B and C).

**Figure 4.**
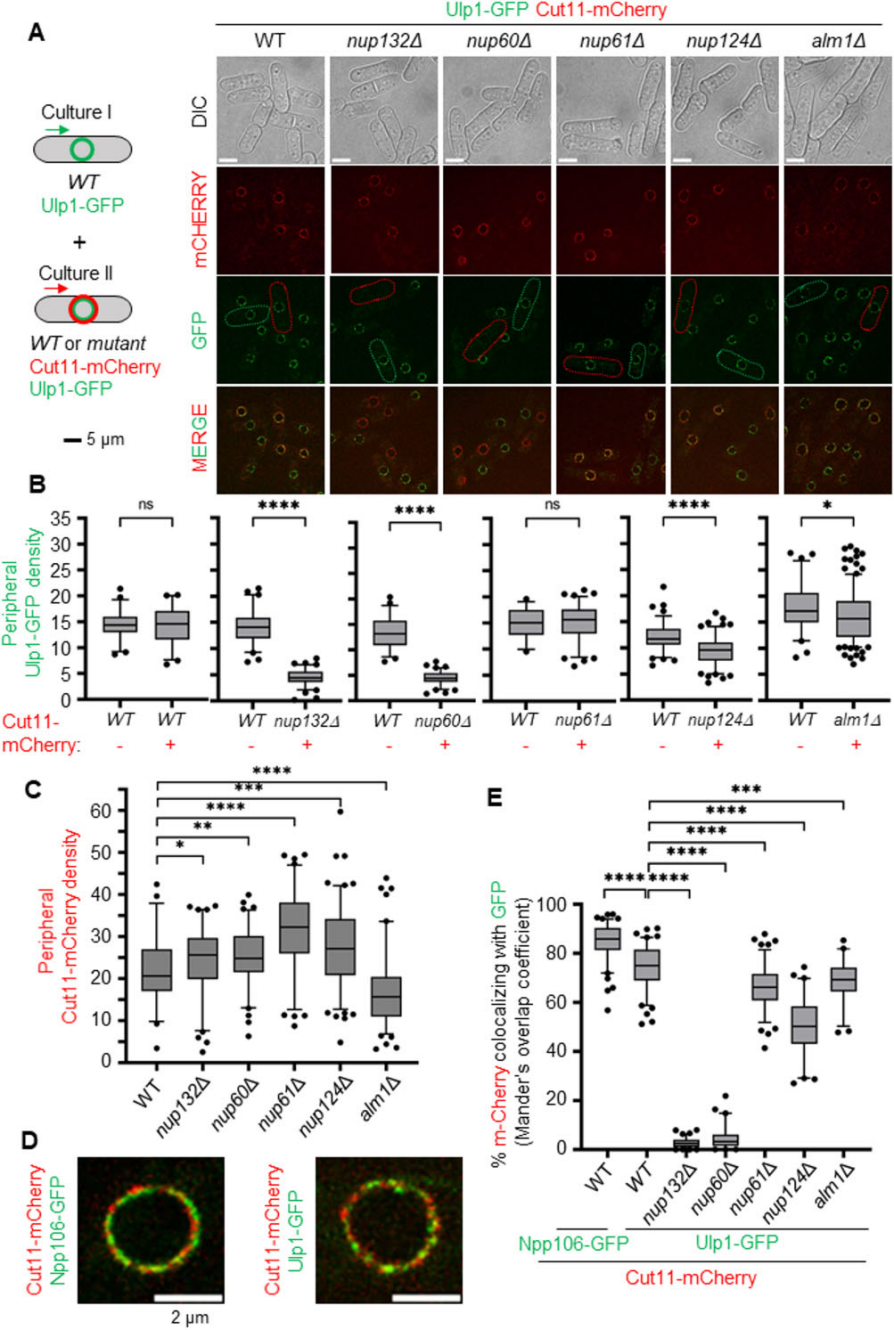
The nuclear basket contributes to sequester the SUMO SENP protease Ulp1 at the nuclear periphery. (**A**) Left panel: scheme of the strategy employed by equally mixing two genetically distinct cell cultures. Right panel: representative cell images of Cut11-mCherry and Ulp1-GFP in indicated strains. Green and red cell borders indicate cells from culture I (expressing Ulp1-GFP) and culture II (expressing Ulp1-GFP Cut11-mCherry), respectively. Scale bar 5 μm. (**B**) Box-and-whisker plots of Ulp1-GFP density (mean fluorescence intensity) at the nuclear periphery in indicated strains and conditions. Boxes represent the 25/75 percentile, black lines indicate the median, the whiskers indicate the 5/95 percentile and dots correspond to minimum and maximum values. *P* value was calculated by Mann–Whitney *U* test (**** *P* ≤ 0.0001; *** *P* ≤ 0.001; ** *P* ≤ 0.01; * *P* ≤ 0.05; ns: non-significant). At least 50 nuclei were analyzed for each strain. (**C**) Box-and-whisker plots of Cut11-mCherry density (mean fluorescence intensity) at the nuclear periphery in indicated strains. Boxes represent the 25/75 percentile, black lines indicate the median, the whiskers indicate the 5/95 percentile and dots correspond to minimum and maximum values. *P* value was calculated by Mann–Whitney *U* test (**** *P* ≤ 0.0001; *** *P* ≤ 0.001; ** *P* ≤ 0.01; * *P* ≤ 0.05; ns: non-significant). At least 50 nuclei were analyzed for each strain. (**D**) Example of the localization of Npp106-GFP and Cut11-mCherry (left panel) or Ulp1-GFP and Cut11-mCherry (right panel) on overlay images. Scale bar: 2 μm. (**E**) Box-and-whisker plots of co-localization between Cut11-mCherry and Ulp1-GFP (Mander's overlap coefficient) in indicated strains. The co-localization between the Npp106-GFP, an inner ring nucleoporin of NPC, and Cut11-mCherry, was performed as a control to show maximum correlation between intensities of those both proteins at the resolution achieved on the images. Boxes represent the 25/75 percentile, black lines indicate the median, the whiskers indicate the 5/95 percentile and dots correspond to minimum and maximum values. *P* value was calculated by Mann–Whitney *U* test (**** *P* ≤ 0.0001; *** *P* ≤ 0.001)

To better characterize the nuclear basket function in sequestrating Ulp1 at the NP, we employed live cell imaging to detect simultaneously Ulp1 in WT and mutant backgrounds and quantify Ulp1 density at the NP. To ensure accuracy, we mixed an equal amount of exponentially growing WT cells expressing Ulp1-GFP with WT or nuclear basket mutants co-expressing Ulp1-GFP and Cut11-mCherry (Figure 4A). This approach allowed us to distinguish WT cells from the mutated strains within the same microscopy field, and thus accurately quantify peripheral Ulp1 irrespective of exposure and acquisition parameters. In addition, as Cut11 is a transmembrane core NPC nucleoporin, we also could quantify the total amount and density of NPCs. As previously reported (42), the nuclear morphology of *alm1Δ* cells was different from WT, with an increase in nuclear perimeter and size (Supplementary Figure S3A and S3B). The total amount of peripheral Ulp1 (i.e. total Ulp1 intensity) decreased in *nup132Δ*, *nup60Δ* and *nup124Δ* cells when compared to WT (Supplementary Figure S3C), resulting in a reduced peripheral Ulp1 density (i.e. intensity normalized by nuclear size, see materials and methods section) (Figure 4A and B). Although the total peripheral Ulp1 intensity was slightly increased in *alm1Δ* cells (Supplementary Figure S3C), the increased nuclear size led to a significant reduction in peripheral Ulp1 density (Figure 4B). The total amount of Cut11 was variable in all strains when compared to WT (Supplementary Figure S3D) but we observed a clear reduction in peripheral Cut11 density in *alm1Δ* cells because of an increased nucleus size (Figure 4C). Finally, we quantified co-localization between Cut11-mCherry and Ulp1-GFP signals as a read-out of Ulp1-associated NPCs, using Manders overlap coefficient (Figure 4D-E) and Pearson correlation coefficient (Supplementary Figure S3E). As a control we first assigned co-localization between Cut11-mCherry and Npp106-GFP, two core components of NPC. Between 80 and 90% of Cut11 signal was associated with Npp106 under our microscopy conditions, validating our methodological approach (Figure 4D-E and Supplementary Figure S3E). In the absence of either Nup132 or Nup60, the low level of Ulp1 appeared to not overlap with Cut11 at the resolution achieved on the images, indicating that Ulp1-associated NPCs are abolished. Despite a lower NPCs density and a reduced Ulp1 expression in the absence of Alm1, Ulp1-associated NPCs were only moderately affected (∼70% compared to ∼75% in the WT background). In contrast, only ∼50% of Cut11 signal was correlated with Ulp1 in *nup124Δ* cells (Figure 4E and Supplementary Figure S3E), indicating that Ulp1-associated NPCs are less abundant. We concluded that Nup60, and to a lesser extent Nup124, are two key components of the nuclear basket that sequester Ulp1 at the NP.

In budding yeast, Mlp1 and Mlp2 act synergistically to sequester Ulp1 to the NP (39). We therefore addressed the role of the second TPR orthologue Nup211, an essential nucleoporin in *S. pombe*. We employed an auxin-inducible degron (AID) approach using the recently developed AID2 version that makes use of OsTIR1-F74A to target AID-tagged proteins for degradation (43). Nup211-HA-mAID was efficiently degraded 30 minutes after the addition of 5-adamantyl-IAA and no degradation was observed in the absence of TIR1-F74A (Supplementary Figure S4A). We observed a ∼40% reduction in Ulp1-GFP expression 60 minutes after 5-adamantyl-IAA addition, compared to the control strain in which TIR1-F74A is not expressed (compare lines 3 and 4 in Supplementary Figure S4B). However, Ulp1-GFP expression was slightly decreased in the strain expressing TIR1-F74A in the absence of 5-adamantyl-IAA (compare lines 1 and 2 in Supplementary Figure S4B). Consistently, these strains showed a significant growth defect when plated on media free of drug (Supplementary Figure S4C), indicating that either the AID2 system applied to Nup211 is leaky or that the C-terminal degron tag partially compromised Nup211 function. When we quantified peripheral Ulp1-GFP by live-cell imaging, we observed that the addition of 5-adamantyl-IAA led to an increased in peripheral Ulp1 density in WT cells and no changes were observed upon degradation of Nup211 (Supplementary Figure S4D). We concluded that Nup211 makes little contribution to Ulp1 expression and peripheral sequestration. We wanted to test the possibility that Alm1 and Nup211 act synergistically to regulate Ulp1 expression and localization, but we failed in generating viable spores combining *alm1* deletion with the *nup211-HA-mAID* allele.

### Tethering of the RFB to Ulp1-associated NPCs rescues RDR defect in *nup60Δ* but not in *alm1Δ cells*

**Figure 5.**
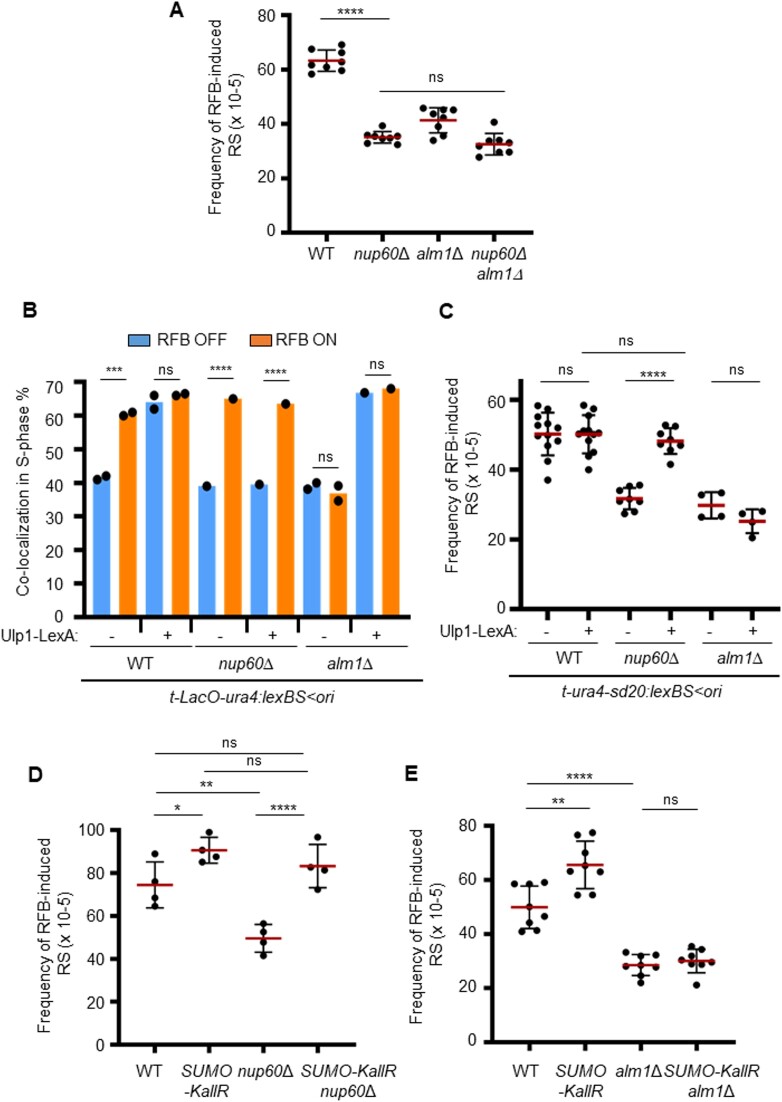
Tethering of Ulp1 to the RFB rescues RDR defect in *nup60Δ* but not *in alm1Δ* cells. (**A**) Frequency of RFB-induced RS in indicated strains and conditions. Dots represent values obtained from independent biological experiments. Red bars indicate mean values ± SD. *P* value was calculated by two-sided *t-*test (**** *P* ≤ 0.0001; ns: non-significant). (**B**) Quantification of co-localization events in S-phase cells in indicated conditions and strains. Dots represent values obtained from independent biological experiments. At least 100 nuclei were analyzed for each strain and condition. *P* value was calculated by two-sided Fisher's exact test (**** *P* ≤ 0.0001; *** *P* ≤ 0.001; ns: non-significant). (**C–E**) Frequency of RFB-induced RS in indicated strains and conditions. Dots represent values obtained from independent biological experiments. Red bars indicate mean values ± SD. *P* value was calculated by two-sided *t-*test (**** *P* ≤ 0.0001; ** *P* ≤ 0.01,* *P* ≤ 0.05 ns: non-significant).

Since Ulp1 is no longer sequestrated at the NP in the absence of Nup60 and the RFB is no longer enriched at the NP in the absence of Alm1, we analyzed the double mutant and found that the level of RFB-induced RS was similar to that observed in *nup60Δ* cells (Figure 5A). This result would be consistent with arrested forks having no access to Ulp1-associated NPCs in the absence of Alm1, resulting in an RDR defect. To directly test this, we employed a previously successful approach to tether Ulp1-LexA to the RFB harboring 8 LexA binding sites (either *t-LacO-ura4:LexBS < ori* for nuclear positioning (Figure 5B) or *t-ura4-sd20:lexA < ori* for RFB-induced RS (Figure 5C)) (15). In WT cells, the *LacO*-marked RFB was constitutively enriched at the NP upon expression of Ulp1-LexA, whatever its activity (OFF or ON), showing that Ulp1 is successfully tethered to the RFB (Figure 5B). Consistent with the role of Nup60 in sequestrating Ulp1 at the NP, the inactive RFB did not shift to the NP in *nup60Δ* cells, but was efficiently enriched at the NP in RFB ON condition. This confirms that Ulp1 is dispensable for the NPC anchorage of the RFB (Figure 5B). By performing chromatin immuno-precipitation (ChIP), we confirmed that Npp106-GFP was similarly enriched at the RFB upon Ulp1-LexA tethering in the RFB ON condition in both WT and *nup60Δ* cells (Supplementary Figure S5). Remarkably, tethering Ulp1-LexA to the active RFB, anchored to NPC, resulted in an increased frequency of RFB-induced RS in *nup60Δ* cells, indicating that the lack of Ulp1-associated NPCs is a limiting step in promoting HR-mediated DNA synthesis (Figure 5C). In addition, we combined the *nup60* deletion with SUMO-KallR, which allows only mono-SUMOylation to occur (cf. Figure 3C). As previously reported (15), we observed a slight increase in RFB-induced RS in SUMO-KallR strain (Figure 5D). As expected, preventing SUMO chain in *nup60Δ* cells restored RFB-induced RS to the WT level, further confirming that the reduction in RDR efficiency caused by defective Ulp1-associated NPCs is alleviated by preventing SUMO chain formation (Figure 5D).

Surprisingly, applying similar approaches to *alm1Δ* cells resulted in different outcomes, indicating a distinct flavor of RDR defect. Preventing SUMO chain formation did not rescue the RDR defect observed in the absence of Alm1 (compare *alm1Δ* and *alm1Δ* SUMO-KallR on Figure 5E), indicating that the RDR defect observed in this mutant is not alleviated by preventing SUMO chain formation. Moreover, tethering Ulp1 to the RFB did not rescue the RDR defect (Figure 5B and C). The analysis of the nuclear positioning of the *LacO*-marked RFB showed that the RFB was efficiently shifted to the NP in *alm1Δ* cells regardless its activity, thus allowing bypassing the role of Alm1 in locating the active RFB at the NP (compare RFB ON condition with or without Ulp1-LexA in *alm1Δ* on Figure 5B). In other words, the artificial anchorage of the RFB to Ulp1-associated NPCs is not sufficient to rescue the RDR defect of *alm1Δ* cells. This indicates that the lack of RFB relocation to the NP is not the underlying cause of the RDR defect and that Alm1 is probably required at NPCs to promote RDR independently of Ulp1. Interestingly, Daga *et al.* have reported that Alm1 is required for proper localization of the proteasome to the NE: several proteasome subunits and anchors, such as Mts2 (also known as Rpt2), Mts4 and Cut8, are not properly localized at the NP in *alm1Δ* cells (42). Furthermore, we previously proposed that the proteasome activity is necessary to promote RDR but that this might not be under Nup132 regulation (15). Given the technical difficulty of restoring a stoichiometric proteasome at the NP in *alm1Δ* cells, we turned our attention to a viable proteasome mutant to address its role in the dynamic of RDR. This decision was also motivated by the fact that the deletion of both *alm1* and *rtf1*, a genetic background needed for Pu-Seq analysis, has been reported to be synthetic lethal (42).

### Proteasome-associated NPCs sustain the dynamics of HR-restarted fork

**Figure 6.**
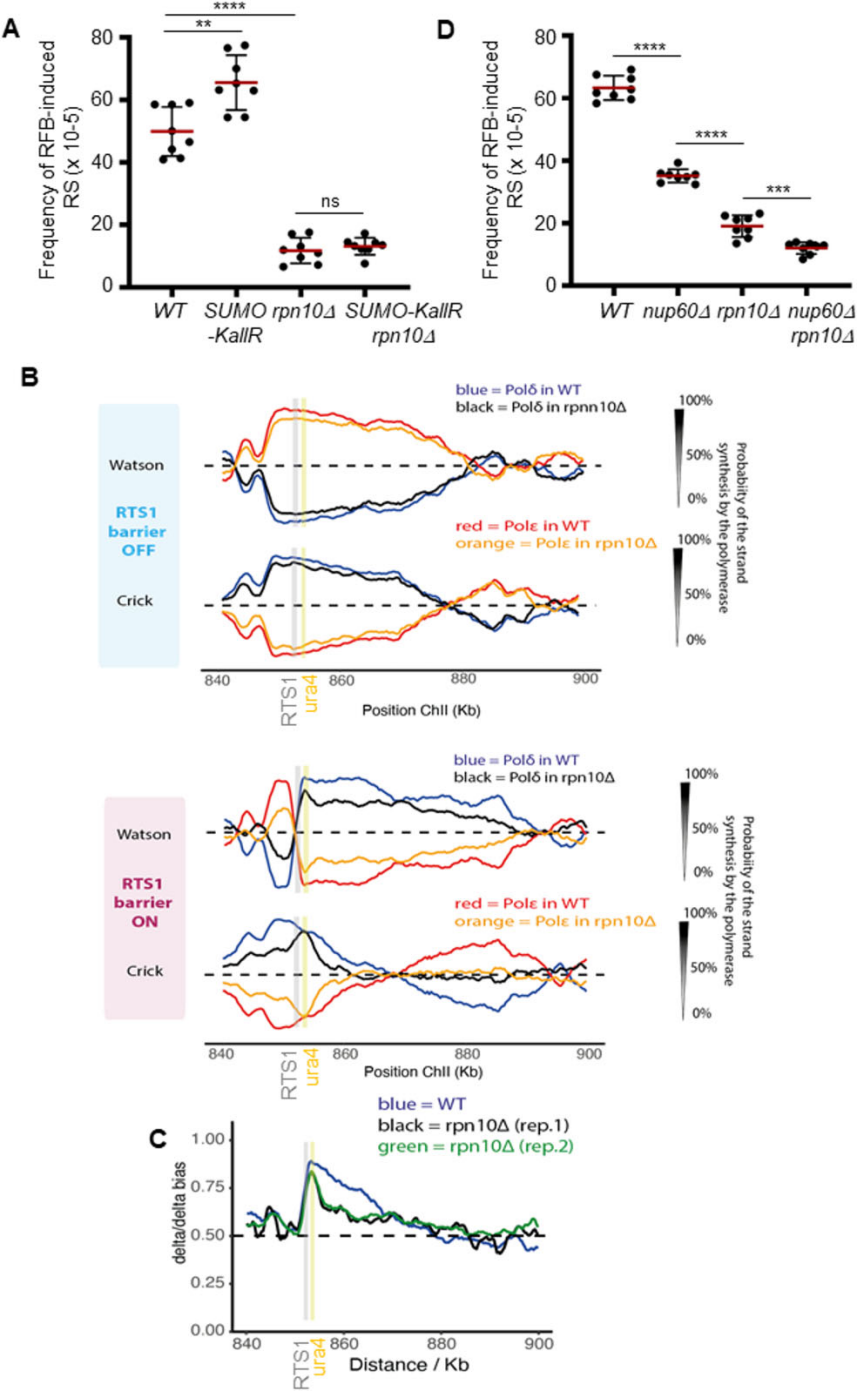
Proteasome-associated NPCs promote the progression of restarted fork. (**A**) Frequency of RFB-induced RS in indicated strains and conditions. Dots represent values obtained from independent biological experiments. Red bars indicate mean values ± SD. *P* value was calculated by two-sided *t*-test (**** *P* ≤ 0.0001; *** *P* ≤ 0.001, ** *P* ≤ 0.01; ns: non-significant). (**B**) Pu-Seq traces of the ChrII locus in *RTS1*-RFB OFF (top panel) and ON (bottom panel) conditions in WT and *rpn10Δ* strains. The usage of Pol delta (in blue and black for WT and *rpn10Δ* cells, respectively) are shown on the Watson and Crick strands. The usage of Pol epsilon (in red and orange for WT and *rpn10Δ* cells, respectively) are shown on the Watson and Crick strands. Note that the switch from Pol epsilon to Pol delta on the Watson strand at the RFB site (gray bar) is indicative of a change in polymerase usage on the leading strand in RFB ON condition. The genomic location of the ARS, the *RTS1*-RFB and the *ura4* marker are indicated by dashed lines, a gray line and a yellow line, respectively. (**C**) Graph of Pol delta/delta bias in RFB ON condition according to chromosome coordinates in WT and two independent replicates of *rpn10Δ* strains. The gray and yellow bars indicate the position of the *RTS*1-RFB and of the *ura4* marker, respectively. (**D**) Frequency of RFB-induced RS in indicated strains and conditions. Dots represent values obtained from independent biological experiments. Red bars indicate mean values ± SD. *P* value was calculated by two-sided *t*-test (**** *P* ≤ 0.0001; *** *P* ≤ 0.001).

Rpn10 is a regulatory subunit of the 19S proteasome that physically interacts with Mts4/Rpn1 and that is enriched at the NP (44–46) and promotes cell resistance to replication blocking agents (Supplementary Figure S6A). We previously reported that, in the absence of Rpn10, the active RFB shifts to the NP but RDR efficiency was severely decreased (15), as evidenced by the strong reduction in RFB-induced RS (Figure 6A). Rpn10 acts as a ubiquitin receptor for the proteasome and its absence results in the accumulation of ubiquitin conjugates. Despite an accumulation of SUMO conjugates in *rpn10Δ* cells (Supplementary Figure S6B), we observed that the defect in RFB-induced RS was not rescued by preventing SUMO chain formation (Figure 6A), a situation similar to the *alm1Δ* mutant. We were unable to investigate whether Alm1 and Rpn10 act in the same pathway to promote RDR as we found that the double mutant is not viable.

To probe the function of Rpn10 in RDR, we applied the Pu-Seq approach to the *rpn10* mutant to compare DNA polymerase usage at, and downstream of, the barrier site. We first noticed that polymerase usage was altered upstream of the RFB specifically in the RFB ON condition in *rpn10Δ* cells when compared to WT (Figure 6B), a phenotype suggestive of loss of integrity of the newly synthetized strand. To test this, we analyzed replication intermediates by 2DGE (as described in Figure 2C and D) and observed that nascent strand degradation at arrested forks was slightly increased in the absence of Rpn10 (Supplementary Figure S6C and D), indicating a role for the proteasome in the negative regulation of fork resection. Based on the Pol δ/δ bias immediately downstream of the RFB, we estimated that, when compared to *rpn10*+ cells, approximately 85% of the expected number of forks were arrested and restarted in *rpn10Δ* cells (Figure 6B and C). Remarkably, the relative change in slope of the Pol δ/δ bias reduction over distance was much steeper in the two replicates from *rpn10Δ* cells when compared to the WT strain, indicating a lower speed or increased instability of the restarted forks (Figure 6C). This slow/unstable replication accounts for the increased number of leftward moving canonical forks evident in the Pu-Seq traces (Figure 6B). We estimated that half of restarted forks progress approximately one third of the distance of WT restarted forks. This scenario contrasts with that observed in the *nup132Δ* cells, in which fewer forks were restarted but the progression of those that did was unaffected. We confirmed that Ulp1 expression and sequestration at the NP were unaffected by the loss of Rpn10 (Supplementary Figure S6E and F). We therefore tested a scenario in which arrested forks are enriched in the NPC environment but devoid of Ulp1 and functional proteasome by analyzing RDR efficiency in *nup60Δ rpn10Δ* cells. We observed an additive effect in RFB-induced RS in this double mutant (Figure 6D), consistent with Rpn10 and Nup60 acting in separate pathways to promote RDR. We concluded that both Ulp1 and the proteasome are required at the NP to foster the dynamics of HR-mediated DNA synthesis by sustaining the efficient initiation of restarted DNA synthesis and the progression of the restarted fork, respectively.

## Discussion

Halted replication forks are diverted to the NP and can associate with NPC components to engage error-free DNA repair pathways (8,14–21). How the NPC environment acts mechanistically to foster the dynamics of DNA repair pathways remains unclear. Here, we reveal that NPCs define a particular nuclear compartment that favors the dynamic of HR-dependent DNA synthesis at dysfunctional forks by two distinct mechanisms. The Ulp1 SUMO protease ensures an efficient initiation of restarted DNA synthesis. This mechanism requires the sequestration of Ulp1 at the NP which is coordinated by the Y complex and the nuclear basket nucleoporin Nup60. The second mechanism relies on the ability of the nuclear basket to enrich proteasome components at the NP (42,46) to foster the progress of restarted DNA polymerases. Surprisingly, preventing SUMO chain formation mitigates the defects in HR-mediated fork restart caused by defective Ulp1-associated NPCs but not the defects caused by defective proteasome-associated NPCs. We thus establish that Ulp1 and the proteasome differentially affect the dynamics of HR-mediated DNA synthesis without compensating for each other.

We previously reported that the Y complex nucleoporin Nup132 promotes RDR in a post-anchoring manner, downstream of Rad51 loading at dysfunctional forks, by sequestrating Ulp1 at the NP (15). Here, we reveal that the nuclear basket contributes to this pathway. Akin to budding yeast (38), the sequestration of Ulp1 at the NP in *S. pombe* requires the nuclear basket nucleoporin Nup60. Despite our efforts, we cannot rule out a synergistic function of TPR homologs, Alm1 and Nup211, in the spatial segregation of Ulp1 at the NP. By mapping DNA polymerase usage during HR-dependent fork restart (28), we establish that Ulp1-associated NPCs are necessary to ensure efficient initiation of restarted DNA synthesis. This function may be linked to the fact that Ulp1 counterbalances the inhibitory effect of SUMO chain on unknown targets. In budding yeast, a similar inhibitory effect of SUMO chain on DNA replication initiation at origins has been reported (47). The MCM helicase and other replication factors were identified as SUMO chain-modified substrates targeted by the SUMO protease Ulp2 and ultimately proteasomal degradation. Although we did not formally address the function of SpUlp2 in RDR, our data clearly highlight a role for Ulp1-associated NPCs in promoting efficient initiation of restarted DNA synthesis. Protein-protein docking studies predicted a higher affinity of SpUlp1 towards SUMO particles compared to ScUlp1, suggesting a more specific role of SpUlp1 in targeting SUMO chain than Ulp2 (48). Alternatively, preventing SUMO chain formation may act on other mechanisms that favor the frequency of replication slippage during the progression of restarted forks. Further investigations are needed to clarify the role played by Pli1-dependent SUMO chain in the dynamics of RDR. Notwithstanding this, our work establishes that the abundance of Ulp1-associated NPCs is not a limiting factor in promoting RDR, as their reduction by 40% in *nup124Δ* cells leads to no noticeable RDR defect.

Our work also establishes that Nup132, a component of the Y complex, and Nup61, a component of the nuclear basket, contribute to the cellular response to replication stress and recovery from transient fork stalling. However, the lack of Ulp1-associated NPCs does not correlate with cell sensitivity to replication-blocking agents or with a defective recovery from HU-stalled forks, since *nup132Δ* and *nup60Δ* cells exhibit distinct phenotype. In addition, no defect in Ulp1-associated NPCs was observed in the absence of Nup61. Therefore, the integrity of the nuclear basket and the Y complex is required to promote cells resistance to replication blocking agents and recovery from HU-stalled forks, beyond the formation of Ulp1-associated NPCs and SUMO homeostasis. This conclusion is consistent with a previous report establishing that NPCs contribute to the DNA damage response, beyond a role in sequestrating Ulp1 at the NP (49). Further investigations are needed to establish, for example, the contribution of macromolecular transport by NPC in the DNA damage response.

We previously reported that the proteasome, whose activity is enriched at the NP (46), promotes RDR in a post-anchoring manner (15). The mapping of DNA polymerase usage during HR-dependent fork restart reveals that a proteasome defect more severely affects the progression of restarted DNA polymerases, with a reduction of forward movement by up to 70%, than the initiation of restarted DNA synthesis. This contrasts with Ulp1 function in contributing primarily to the initiation of DNA synthesis with no apparent contribution to the dynamics progression of restarted DNA polymerases. This division of labour between the proteasome and the SUMO protease in ensuring the dynamics of HR-dependent fork restart is reinforced by the fact that these activities cannot compensate for each other. Indeed, the artificial tethering of the RFB to NPCs in the *alm1Δ* mutant shows that Ulp1-associated NPCs are insufficient to promote efficient RDR without a functional proteasome at the NP. We noticed that RFB-induced RS are more severely decreased in the absence of Rpn10 than in the absence of Alm1. This might be due to a more drastic effect of the loss of Rpn10 on proteasome functionality than the loss of Mts2/Rpt2 and Mts4, as observed in *alm1Δ* cells. We were unable to test RDR efficiency in the double *rpn10Δ alm1Δ* mutant to address epistatic interaction since this genetic background is not viable. This finding is consistent with a previous report, showing that the deletion of both *rpn10* and *mts4* results in synthetic lethality (44). Alternatively, the extensive degradation of nascent strand at arrested forks observed in *rpn10Δ* cells, but not in *alm1Δ* cells, may contribute to the more severe defect in RDR efficiency. Additionally, the fact that only the NP-enriched fraction of the proteasome is defective in the absence of Alm1 could contribute to a less severe defect in RDR efficiency.

Our genetic analysis establishes that defect in fostering the progress of restarted DNA synthesis caused by defective proteasome-associated NPCs cannot be alleviated by preventing SUMO chain, contrasting with the defect caused by Ulp1-associated NPCs. This suggests distinct specificities between the proteasome and Ulp1 towards SUMOylated targets which affect differentially the dynamics resumption of DNA synthesis at dysfunctional forks. We do not exclude that SUMO-independent poly-ubiquitination, targeted by Rpn10 for proteasomal degradation, plays a role in promoting RDR. However, we previously identified that the SUMO Targeted Ubiquitin Ligase (STUbL) Slx8-Rfp1-Rfp2, a family of E3 ubiquitin ligases, that targets SUMOylated proteins for degradation (50), promotes both the relocation of dysfunctional forks to NPCs and RDR efficiency in a Pli1-dependent manner (15). This supports the notion that mono-SUMOylated or chain-free multi-SUMOylated factors are potential targets of a proteasome and Slx8-dependent pathway that ensures the progress of restarted DNA polymerases. SUMO chain-independent functions of STUbLs are documented, including the relocation of forks collapsed at CAG repeats via mono-SUMOylation recognized by the SUMO interacting motif of ScSlx5 (17). The human STUbL RNF4 can also bind the substrate ETV4 mono-SUMOylated on multiple lysines, in a process requiring the multiple SIM domains of RFN4 (51).

Our work also identified that, in the absence of the nuclear basket nucleoporin Alm1, the RFB was no longer enriched at the NP. To our knowledge, TPR homologs have not been previously implicated in anchoring DNA lesions to NPCs in yeast models. Upon telomeric replication stress, human telomeres relocate to the NP and associate with NPC components, including TPR, to resolve replication defects (19). Depletion of human TPR is associated with a variety of replication defects and TPR was proposed to coordinate at NPCs a network of factors involved in RNA metabolism to protect cells from RNA-mediated replication stress (52). Given the nuclear morphology alterations in the absence of Alm1, we cannot exclude that the lack of anchorage is an indirect effect. In human cells, the mobility of stressed forks towards the NP requires F-nuclear actin polymerization (8,20), but such a mechanism has not been reported in yeast. We estimated that, in the absence of Alm1, the RFB must explore a nuclear volume 40% larger to reach the NP and associate with NPCs whose abundance is reduced by one quarter.

Overall, this work uncovers two mechanisms by which the NPC environment ensures the dynamic of HR-dependent replication restart, streamlining the need for dysfunctional forks to change nuclear positioning. Ulp1-associated NPCs contribute to the efficient initiation of restarted DNA synthesis to engage a Polδ/Polδ DNA synthesis, whereas proteasome-associated NPCs foster the progression of restarted DNA synthesis. These two functions cannot compensate for each other, are differently required and ensured by distinct components of the NPC.

## Supplementary Material

gkae526_Supplemental_File

## Data Availability

The data underlying this article are available in Mendeley data and are available at ‘Schirmeisen, Naiman et al 2023’, Mendeley Data, V1, at doi: 10.17632/2kgnb9d66r.1. RAW data from Pu-Seq experiments are available under GEO dataset GSE247371. All relevant data are available and further information and requests for reagents and resources should be directed to and will be fulfilled by Dr. Sarah A.E. Lambert (sarah.lambert@curie.fr).
