# Supplementary material for: SUMO protease and proteasome recruitment at the nuclear periphery differently affect replication dynamics at arrested forks
Source: Nucleic Acids Res. 2024 Jun 25;52(14):8286–302. doi: 10.1093/nar/gkae526 (PMC11317160; doi:10.1093/nar/gkae526)
Supplement: gkae526_Supplemental_File [file gkae526_supplemental_file.pdf]

SUMO protease and proteasome recruitment at the nuclear periphery differently affect replication dynamics at arrested forks.

Kamila Schirmeisen[1,2,#], Karel Naiman[3,4,#], Karine Fréon[1,2], Laetitia Besse[5], Shrena Chakraborty[1,2], Anissia Ait Saada[1,2], Antony M. Carr[4], Karol Kramarz[6,*] and Sarah AE Lambert[1,2,7,*]

Supplemental Figures

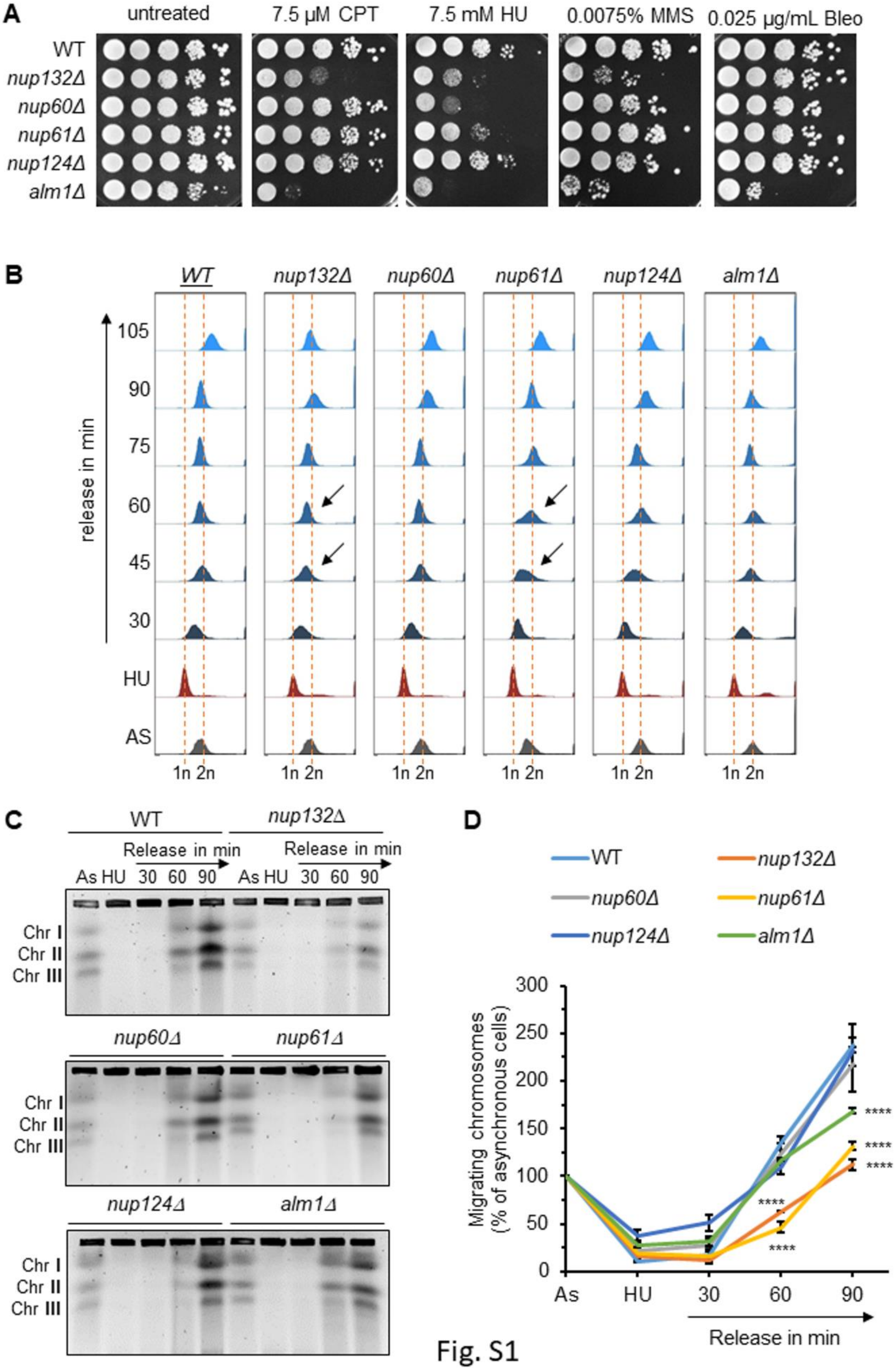
A
untreated
7.5 µM CPT
7.5 mM HU
0.0075% MMS
0.025 µg/mL Bleo
WT
nup132Δ
nup60Δ
nup61Δ
nup124Δ
alm1Δ
B
WT
nup132Δ
nup60Δ
nup61Δ
nup124Δ
alm1Δ
release in min
105
90
75
60
45
30
HU
AS
1n 2n
C
WT
nup132Δ
Release in min
As HU 30 60 90 As HU 30 60 90
Chr I
Chr II
Chr III
nup60Δ
nup61Δ
nup124Δ
alm1Δ
D
WT
nup132Δ
nup60Δ
nup61Δ
nup124Δ
alm1Δ
Migrating chromosomes (% of asynchronous cells)
0
50
100
150
200
250
300
As
HU
30
60
90
Release in min
****

Fig. S1

Figure S1: Role of the nuclear basket in the recovery from HU-induced stalled forks.

A. Sensitivity of indicated strains to indicated genotoxic drugs. Ten-fold serial dilutions of exponential cultures were dropped on appropriate plates. Bleo: bleomycin; CPT: camptothecin; HU: hydroxyurea; MMS: methyl methane sulfonate.

B. Flow cytometry analysis of indicated strains in indicated conditions. Logarithmically growing cells (AS: Asynchronous cells) were exposed to 20 mM HU for 4 hours (HU time point) and then released into fresh, HU-free, rich medium YES at 30°C to monitor S-phase progression at the indicated time after release. Arrows indicate the delay in S phase progression compared to *WT*.

C. Analysis of chromosomes by pulse field gel electrophoresis (PFGE) in the above-mentioned conditions (as in b). Representative images of chromosome migration during PFGE in indicated strains and conditions.

D. Quantification of % of chromosomes migrating into the gel after release from HU block. Values are means of two independent biological replicates ± standard deviation (SD). *p* value was calculated by two-sided Fisher's exact test (**** $p \leq 0.0001$).

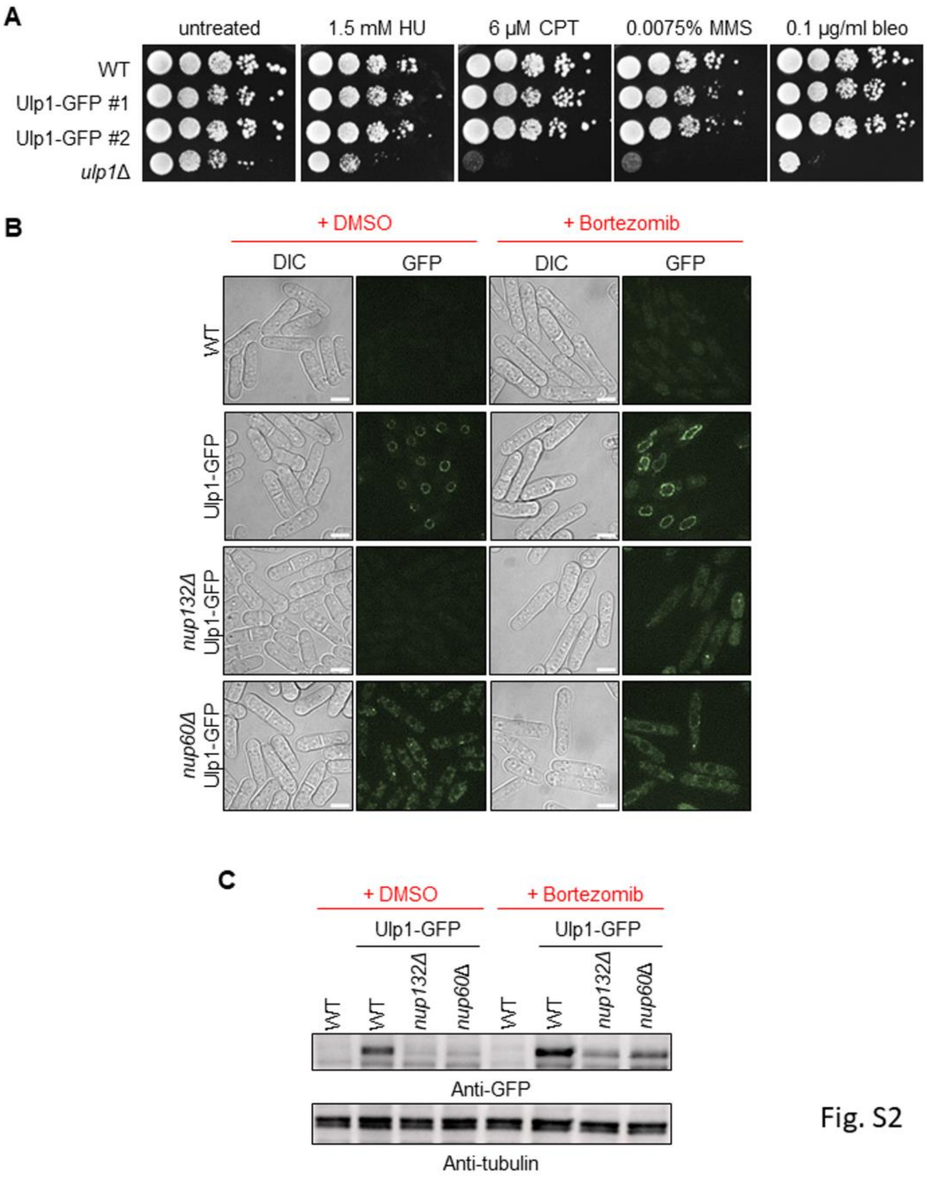

Fig. S2

Figure S2: The downregulation of Ulp1 expression is caused by the proteasome.

A. Ulp1-GFP is a functional fusion protein. Ten-fold serial dilutions of exponential cultures were dropped on appropriate plates. Bleo: bleomycin; CPT: camptothecin; HU: hydroxyurea; MMS: methyl methane sulfonate.

B. Cell imaging of Ulp1-GFP in indicated strains and conditions. Representative cell images of Ulp1-GFP in indicated strains in presence or absence of bortezomib. Scale bar: 5μm.

C Expression of Ulp1-GFP in indicated strains and conditions. An untagged WT strain was included as a control for antibody specificity. Tubulin was used as a loading control.

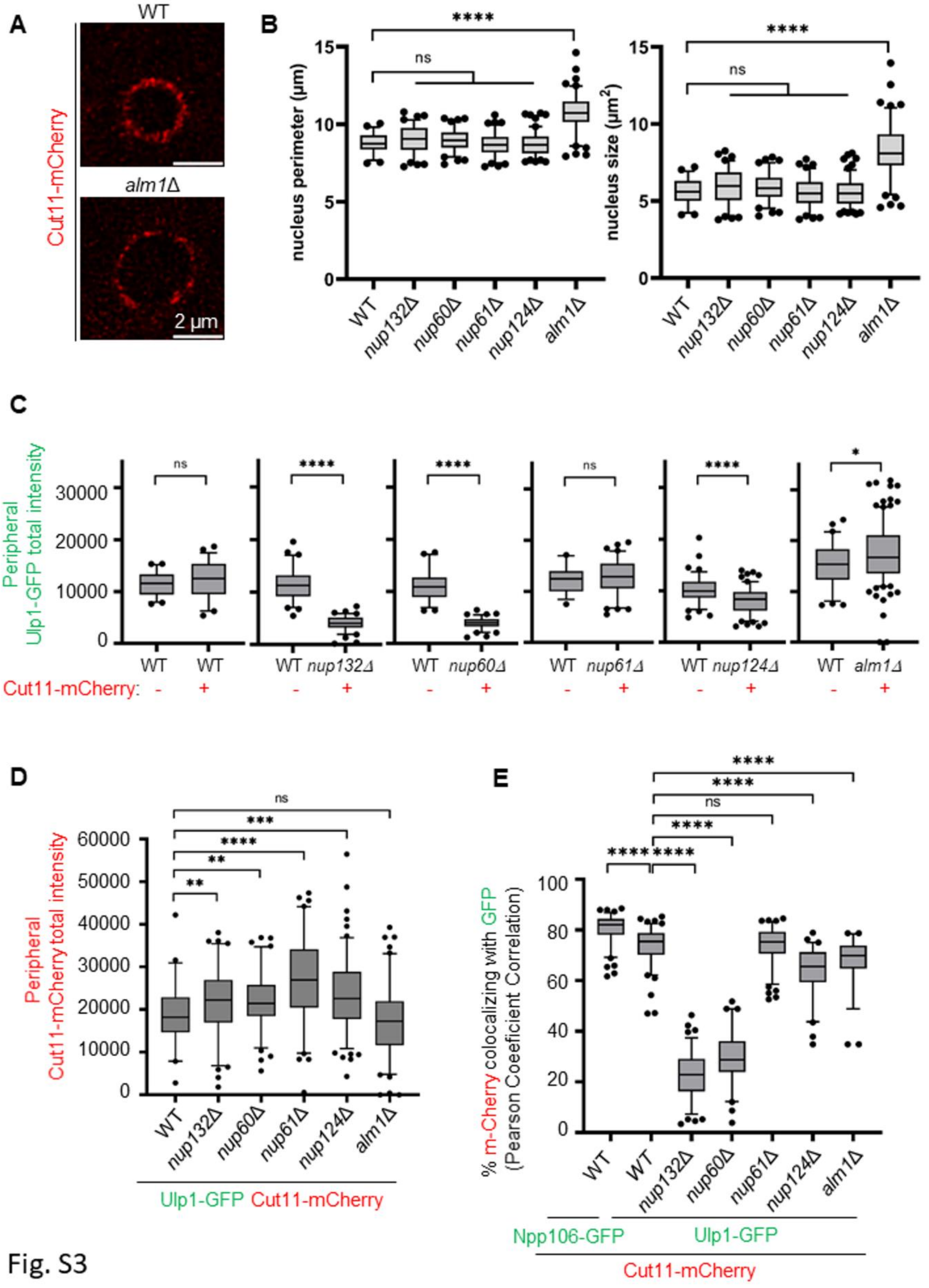
A
WT
Cut11-mCherry
alm1Δ
2 μm
B
nucleus perimeter (μm)
nucleus size (μm²)
ns
****
WT
nup132Δ
nup60Δ
nup61Δ
nup124Δ
alm1Δ
C
Peripheral
Ulp1-GFP total intensity
Cut11-mCherry:
D
Peripheral
Cut11-mCherry total intensity
Ulp1-GFP Cut11-mCherry
E
% m-Cherry colocalizing with GFP
(Pearson Coeficient Correlation)
Npp106-GFP
Ulp1-GFP
Cut11-mCherry

Fig. S3

Figure S3: Image quantification of nuclear morphology parameters, Ulp1-GFP and Cut11-mCherry intensity.

A. Example of fluorescence images of nuclei in cells expressing the endogenous Cut11-mCherry fusion protein in indicated strains. Scale bare 2 µm.

B. Box-and-whisker plots of nucleus perimeter (left panel) and nucleus size (right panel) in indicated strains. Boxes represent the 25/75 percentile, black lines indicate the median, the whiskers indicate the 5/95 percentile and dots correspond to minimum and maximum values. *p* value was calculated by Mann-Whitney U test (**** $p \leq 0.0001$; ns: non-significant). At least 50 nuclei were analyzed for each strain.

C. Box-and-whisker plots of Ulp1-GFP total intensity (raw integrated density) at the nuclear periphery in indicated strains and conditions. Boxes represent the 25/75 percentile, black lines indicate the median, the whiskers indicate the 5/95 percentile and dots correspond to minimum and maximum values. *p* value was calculated by Mann-Whitney U test (**** $p \leq 0.0001$; * $p \leq 0.05$; ns: non-significant). At least 50 nuclei were analyzed for each strain.

D. Box-and-whisker plots of Cut11-mCherry total intensity (raw integrated density) at the nuclear periphery in indicated strains and conditions. Boxes represent the 25/75 percentile, black lines indicate the median, the whiskers indicate the 5/95 percentile and dots correspond to minimum and maximum values. *p* value was calculated by Mann-Whitney U test (**** $p \leq 0.0001$; *** $p \leq 0.001$; ** $p \leq 0.01$; ns: non-significant). At least 50 nuclei were analyzed for each strain.

E. Box-and-whisker plots of co-localization between Cut11-mCherry and Ulp1-GFP (using the Pearson correlation coefficient) in indicated strains. The co-localization between the Npp106-GFP, an inner ring nucleoporin of NPC, and Cut11-mCherry, was performed as a control to show maximum correlation between intensities of both proteins at the resolution achieved on the images. Boxes represent the 25/75 percentile, black lines indicate the median, the whiskers indicate the 5/95 percentile and dots correspond to minimum and maximum values. *p* value was calculated by Mann-Whitney U test (**** $p \leq 0.0001$; ns: non-significant). At least 50 nuclei were analyzed for each strain.

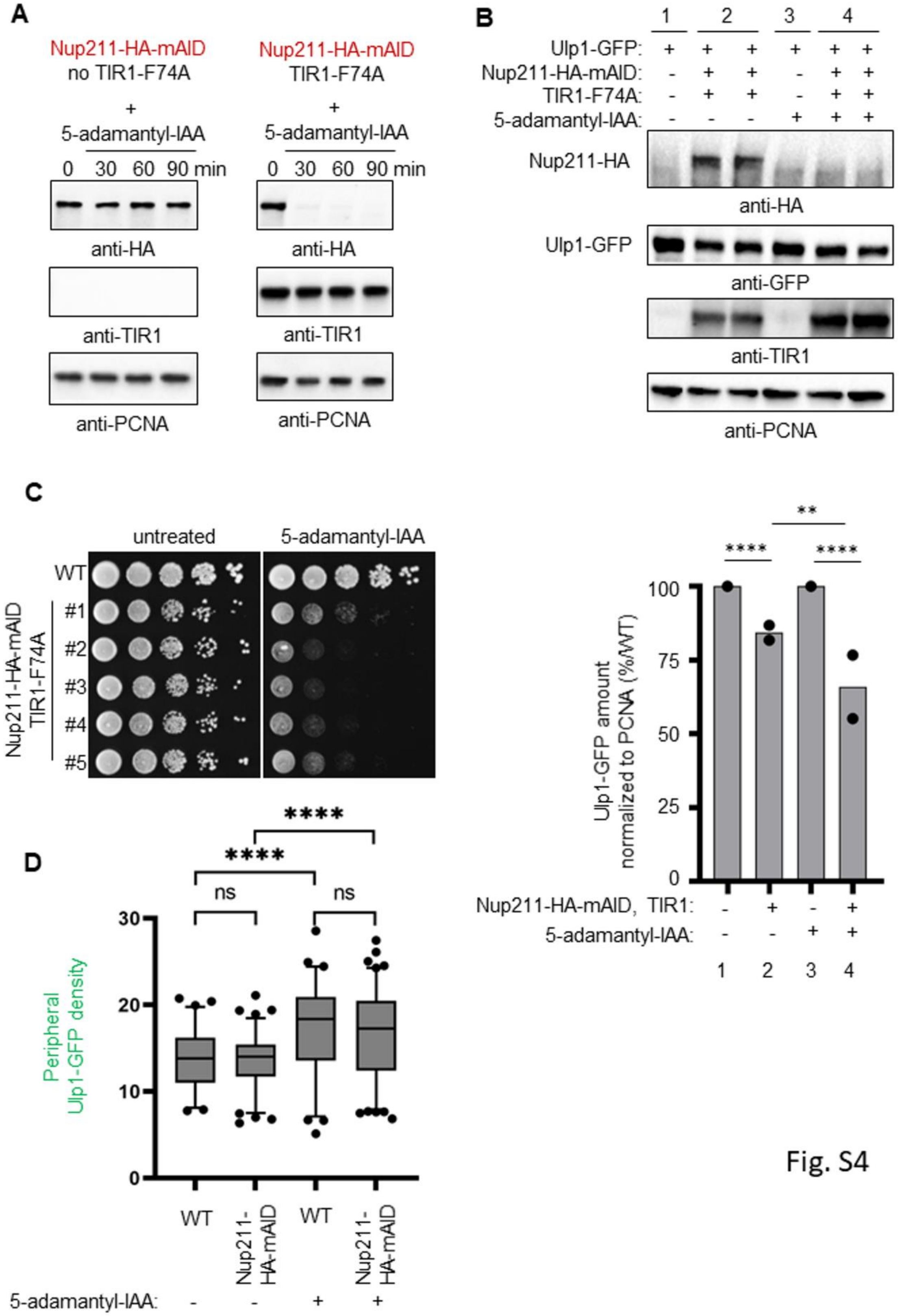
A
Nup211-HA-mAID
no TIR1-F74A
+
5-adamantyl-IAA
0 30 60 90 min
anti-HA
anti-TIR1
anti-PCNA
Nup211-HA-mAID
TIR1-F74A
+
5-adamantyl-IAA
0 30 60 90 min
anti-HA
anti-TIR1
anti-PCNA
B
1 2 3 4
Ulp1-GFP: + + + + + +
Nup211-HA-mAID: - + + - + +
TIR1-F74A: - + + - + +
5-adamantyl-IAA: - - - + + +
Nup211-HA
anti-HA
Ulp1-GFP
anti-GFP
anti-TIR1
anti-PCNA
C
untreated
5-adamantyl-IAA
WT
#1
#2
#3
#4
#5
Nup211-HA-mAID TIR1-F74A
**
****
****
Ulp1-GFP amount normalized to PCNA (%/WT)
100
75
50
25
0
Nup211-HA-mAID, TIR1: - + - +
5-adamantyl-IAA: - - + +
1 2 3 4
D
****
****
ns
ns
Peripheral Ulp1-GFP density
30
20
10
0
WT
Nup211-HA-mAID
WT
Nup211-HA-mAID
5-adamantyl-IAA: - - + +

Fig. S4

Figure S4: An auxin-induced degron approach to conditionally downregulate Nup211.

A. Expression of Nup211-HA-mAID fusion protein in strains expressing TIR1-F74A (right panels) or not (left panels) as a function of time (in minute) upon addition of 5-adamentyl-IAA. PCNA was used as a loading control.

B. Top panels: expression of Ulp1-GFP, Nup211-HA-mAID and TIR1 in indicated conditions. PCNA was used as loading control. Bottom panel: quantification. Dots represent values obtained from independent biological experiments. The normalized amount of Ulp1 was calculated by dividing the GFP signal by PCNA. The normalized amount of Ulp1-GFP in mutants was indicated as a percentage of the *WT*. *p* value was calculated by two-sided Fisher's exact test (**** $p \leq 0.0001$; ** $p \leq 0.01$).

C. Cell growth assay of indicated strains. Ten-fold serial dilutions of exponential cultures were dropped on plates containing 5-adamantyl-IAA (right panel) or not (left panel). Five independent clones expressing Nup211-HA-mAID were tested alongside the *WT* strain. Note the cell growth defect of Nup211-HA-mAID strains in the absence of 5-adamantyl-IAA is indicative of a lack of Nup211 functionality.

D. Box-and-whisker plots of Ulp1-GFP density (mean fluorescence intensity) at the nuclear periphery in indicated strains and conditions. Boxes represent the 25/75 percentile, black lines indicate the median, the whiskers indicate the 5/95 percentile and dots correspond to minimum and maximum values. *p* value was calculated by Mann-Whitney U test (**** $p \leq 0.0001$; ns: non-significant). At least 50 nuclei were analyzed for each strain.

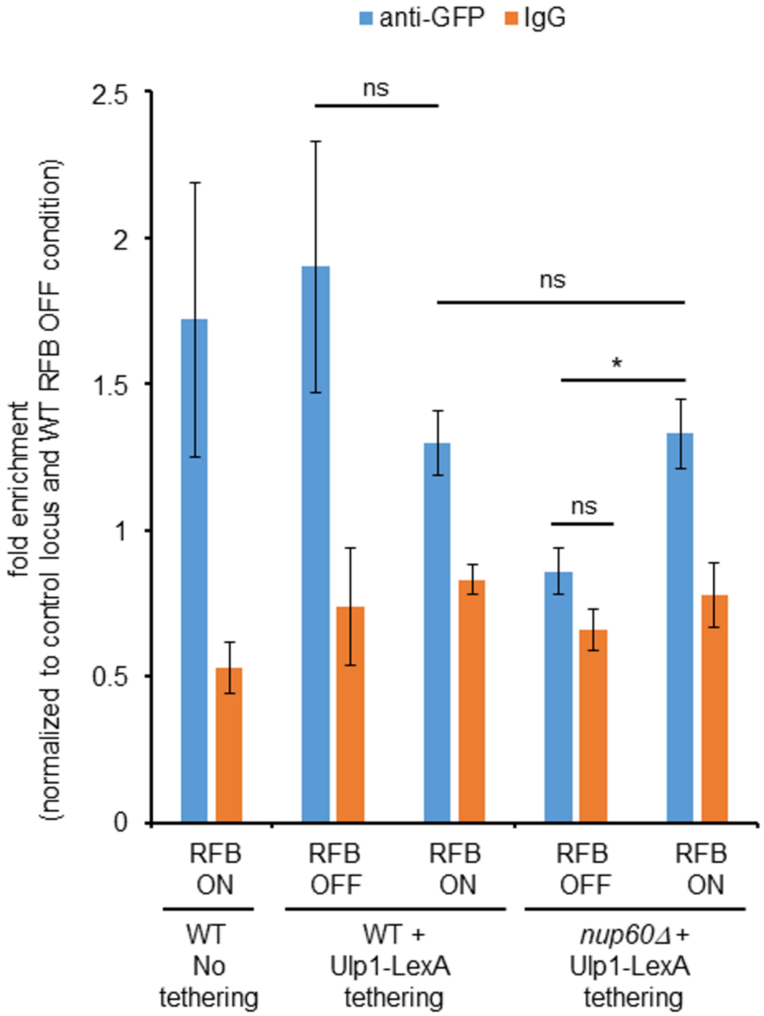

Figure S5: Artificial targeting of Ulp1-LexA to LexA binding sites-containing RFB allows the tethering of the RFB to NPC in wild type and *nup60Δ* cells.

The binding of Npp106-GFP to the RFB was analysed by chromatin immuno-precipitation (ChIP) followed by qPCR analysis using either anti-GFP antibody or IgG as control in indicated strains and conditions. Starting quantities were normalized to a control and unrelated locus and to Wild type RFB Off condition, in the absence of tethering. Values are the mean of 3 to 4 independent biological replicates, errors bars correspond to stander error of the mean (sem). Statistical difference was detected between the samples using the two-sided *t*-test. * $p<0.05$.

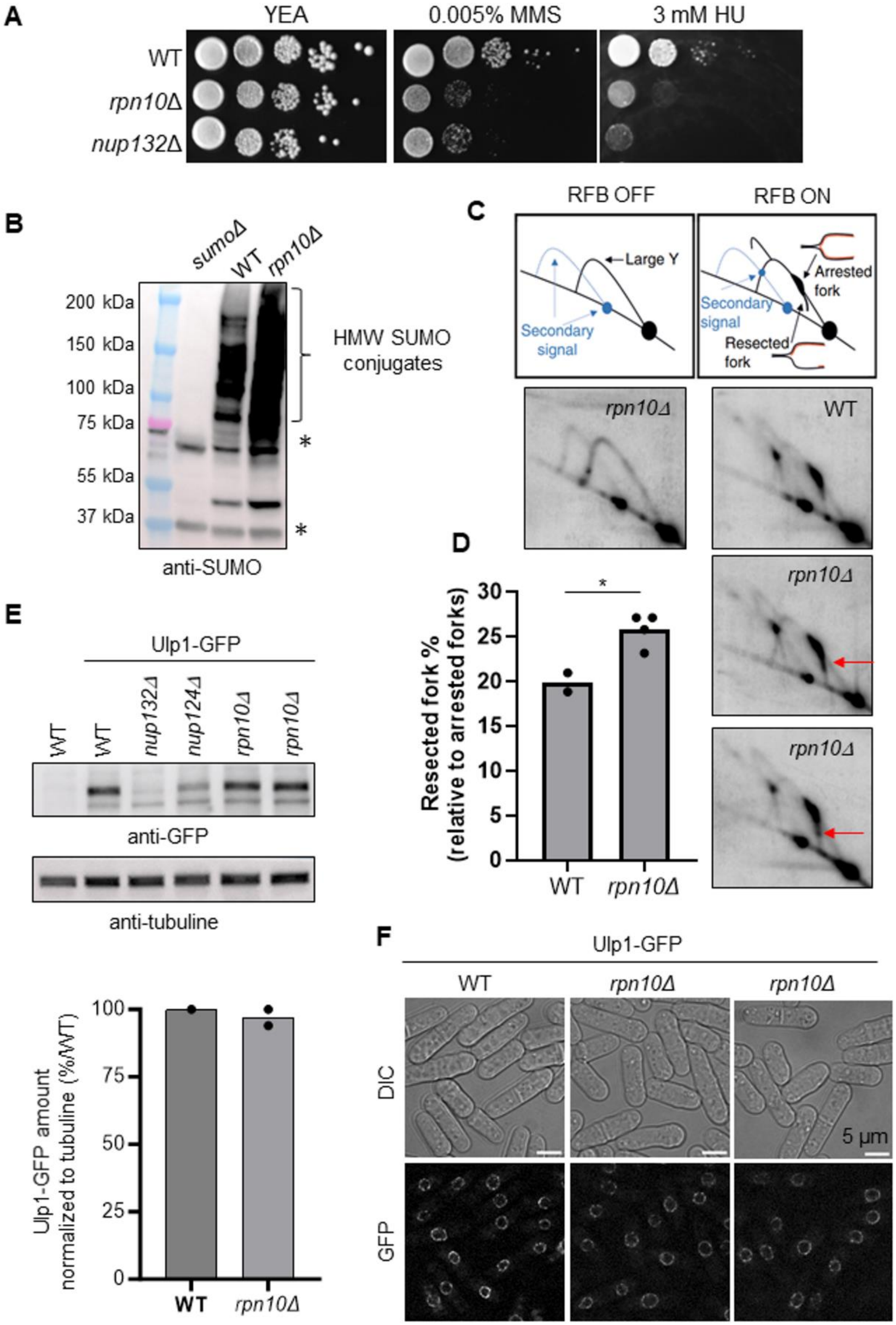
A
YEA
0.005% MMS
3 mM HU
WT
rpn10Δ
nup132Δ
B
sumoΔ
WT
rpn10Δ
200 kDa
150 kDa
100 kDa
75 kDa
55 kDa
37 kDa
HMW SUMO conjugates
*
*
anti-SUMO
C
RFB OFF
RFB ON
Large Y
Secondary signal
Arrested fork
Secondary signal
Resected fork
rpn10Δ
WT
rpn10Δ
rpn10Δ
D
Resected fork % (relative to arrested forks)
*
WT
rpn10Δ
E
Ulp1-GFP
WT
WT
nup132Δ
nup124Δ
rpn10Δ
rpn10Δ
anti-GFP
anti-tubuline
Ulp1-GFP amount normalized to tubuline (%/WT)
WT
rpn10Δ
F
Ulp1-GFP
WT
rpn10Δ
rpn10Δ
DIC
GFP
5 µm

Fig. S6

Figure S6: Characterization of *rpn10* mutant.

A. The absence of Rpn10 results in cell sensitivity to replication blocking agents. Ten-fold serial dilutions of exponential cultures were dropped on appropriate plates. HU: hydroxyurea; MMS: methyl methane sulfonate.

B. Expression of SUMO conjugates in indicated strains. A strain deleted for *pmt3* gene that encodes the SUMO polypeptide (*sumoΔ*) was added as control for antibody specificity. * indicates unspecific signal.

C. Top panel: scheme of replication intermediates (RI) analyzed by neutral-neutral 2DGE of the *AseI* restriction fragment in RFB OFF and ON conditions. Partial restriction digestion caused by psoralen-crosslinks results in a secondary arc indicated on scheme by blue dashed lines. Bottom panels: representative RI analysis in indicated strains and conditions. The *ura4* gene was used as a probe. The red arrow indicates the tail signal resulting from resected forks.

D. Quantification of resected forks in indicated strains. Dots represent values obtained from independent biological experiments. Statistical difference was detected between the samples using the two-sided *t*-test. * $p<0.05$.

E. Top panel: expression of Ulp1-GFP in indicated strains. An untagged *WT* strain was included as control for antibody specificity. Tubulin was used as a loading control. Bottom panel: quantification. The normalized amount of Ulp1 was calculated by dividing the GFP signal by tubulin signal. The normalized amount of Ulp1-GFP in the mutants is indicated as a percentage of the *WT*. Dots represent values obtained from independent biological experiments.

F. Example of fluorescence (bottom panel) and bright-field images (top panel, DIC) of cells expressing the endogenous Ulp1-GFP fusion protein in indicated strains. Scale bare 5 µm.

Supplementary Table 1: strain list

Strain number	Mating type	Genotype	Reference
**KK1467**	*h-*	*LoxP-cdc6-L591G-LoxM3 rtf1::Nat loxP-rnh201RED:KAN-loxM3 ChrII-8535:Rura RTS1::phleo ura4-D18 ade6-704 leu1-32*	this study
**KK1470**	*h-*	*LoxP-cdc6-L591G-LoxM3 Nat:ADH1:rtf1 loxP-rnh201-RED:Kan-loxM3 ChrII-8535:Rura RTS1::phleo ura4-D18 ade6-704 leu1-32*	this study
**KK1473**	*h-*	*loxP-cdc20-M630F-loxM3 rtf1::Nat loxP-rnh201RED:KAN-loxM3 ChrII-8535:Rura RTS1::phleo ura4-D18 ade6-704 leu1-32*	this study
**KK1475**	*h-*	*loxP-cdc20-M630F-loxM3 Nat:ADH1:rtf1 loxP-rnh201RED:KAN-loxM3 ChrII-8535:Rura RTS1::phleo ura4-D18 ade6-704 leu1-32*	this study
**KK1899**	*h-*	*nup132::Hygro LoxP-cdc6-L591G-LoxM3 rtf1::Nat loxP-rnh201RED:KAN-loxM3 ChrII-8535:Rura RTS1::phleo ura4-D18 ade6-704 leu1-32*	this study
**KK1901**	*h-*	*nup132::Hygro LoxP-cdc6-L591G-LoxM3 Nat:ADH1:rtf1 loxP-rnh201RED:KAN-loxM3 ChrII-8535:Rura RTS1::phleo ura4-D18 ade6-704 leu1-32*	this study
**KK1903**	*h-*	*nup132::Hygro loxP-cdc20-M630F-loxM3 rtf1::Nat loxP-rnh201RED:KAN-loxM3 ChrII-8535:Rura RTS1::phleo ura4-D18 ade6-704 leu1-32*	this study
**KK1905**	*h-*	*nup132::Hygro loxP-cdc20-M630F-loxM3 Nat:ADH1:rtf1 loxP-rnh201RED:KAN-loxM3 ChrII-8535:Rura RTS1::phleo ura4-D18 ade6-704 leu1-32*	this study
**KK1377**	*h+*	*ade6-704 leu1-32 ura4-D18*	this study
**KK1557**	*h+*	*nup132::Nat ade6-704 leu1-32 ura4-D18*	this study
**KK1561**	*h+*	*nup60::Hygro ade6-704 leu1-32 ura4-D18*	this study
**KK1578**	*h-*	*nup61::Hygro ade6-704 leu1-32 ura4-D1*	this study
**KK1599**	*h-*	*nup124::Hygro ade6-704 leu1-32 ura4-D18*	this study
**KK1384**	*h+*	*alm1::Hygro ade6-704 leu1-32 ura4-D18*	this study
**KK1707**	*h+*	*nmt41:rtf1:sup35 ade6-704 leu1-32 t- ura4-SD20<ori (uraR)*	this study
**KK931**	*h+*	*nup60::Hygro nmt41:rtf1:sup35 ade6-704 leu1-32 t- ura4-SD20<ori (uraR)*	this study
**KK953**	*h+*	*nup61::Hygro nmt41:rtf1:sup35 ade6-704 leu1-32 t- ura4-SD20<ori (uraR)*	this study
**KK1593**	*h+*	*nup124::Hygro nmt41:rtf1:sup35 ade6-704 leu1-32 t- ura4-SD20<ori (uraR)*	this study
**KK2556**	*h-*	*rpn10::Hygro nmt41:rtf1:sup35 ade6-704 leu1-32 t- ura4-SD20<ori (uraR)*	this study

**KK2564**	*h-*	*rpn10::Hygro nup60::Hygro nmt41:rtf1:sup35 ade6-704 leu1-32 t- ura4-SD20<ori (uraR)*	this study
**KK2576**	*h-*	*alm1::Hygro nmt41:rtf1:sup35 ade6-704 leu1-32 t- ura4-SD20<ori (uraR)*	this study
**KK2568**	*h+*	*alm1::Hygro nup60::Hygro nmt41:rtf1:sup35 ade6-704 leu1-32 t- ura4-SD20<ori (uraR)*	this study
**KK300**	*h+*	*npp106-GFP:Nat arg3::mCherry-LacI nmt41:rtf1:sup35 ade6-704 leu1-32 t-LacO 7,9Kb:Kan:*$ura4^{+}$*<ori (uraR)*	Kramarz et al., 2020
**KK301**	*h+*	*nup60::Hygro npp106-GFP:Nat arg3::mCherry-LacI nmt41:rtf1:sup35 ade6-704 leu1-32 t-LacO 7,9Kb:Kan:*$ura4^{+}$*<ori (uraR)*	this study
**KK166**	*h-*	*nup61::Hygro npp106-GFP:Nat arg3::mCherry-LacI nmt41:rtf1:sup35 ade6-704 leu1-32 t-LacO 7,9Kb:Kan:*$ura4^{+}$*<ori (uraR)*	this study
**KK32**	*h+*	*nup124::Hygro npp106-GFP:Nat arg3::mCherry-LacI nmt41:rtf1:sup35 ade6-704 leu1-32 t-LacO 7,9Kb:Kan:*$ura4^{+}$*<ori (uraR)*	this study
**KK1580**	*h-*	*alm1::Hygro npp106-GFP:Nat arg3::mCherry-LacI nmt41:rtf1:sup35 ade6-704 leu1-32 t-LacO 7,9Kb:Kan:*$ura4^{+}$*<ori (uraR)*	this study
**KK687**	*h-*	*ulp1::Kan nmt41:rtf1:sup35 ade6-704 leu1-32 t-ura4-SD20<ori (uraR)*	this study
**KK1553**	*h-*	*ulp1-GFP:Kan ade6-704 leu1-32 ura4-D18*	this study
**KK2590**		*ulp1-GFP:Kan rpn::hygro ade6-704 leu1-32 ura4-D18*	this study
**KK1555**	*h-*	*nup132::Nat ulp1-GFP:Kan ade6-704 leu1-32 ura4-D18*	this study
**KK1560**	*h-*	*nup60::Hygro ulp1-GFP:Kan ade6-704 leu1-32 ura4-D18*	this study
**KK1575**	*h+*	*nup61::Hygro ulp1-GFP:Kan ade6-704 leu1-32 ura4-D18*	this study
**KK1596**	*h+*	*nup124::Hygro ulp1-GFP:Kan ade6-704 leu1-32 ura4-D18*	this study
**KK1996**	*h+*	*alm1::Hygro ulp1-GFP:Kan ade6-704 leu1-32 ura4-D18*	this study
**KK1562**	*h-*	*pmt3 ::Kan-ura4 nmt41:rtf1:sup35 ade6-704 leu1-32 t- ura4-SD20<ori (uraR)*	this study
**KK2018**	*h+*	*SUMO-KallR (pmt3-KallR) ade6-704 leu1-32 ura4-D18*	this study
**KK1965**	*h-*	*ulp1-GFP:Kan cut11-mCherry:Hygro ade6-704 leu1-32 ura4-D18*	this study
**KK1967**	*h+*	*nup132::Nat ulp1-GFP:Kan cut11-mCherry:Hygro ade6-704 leu1-32 ura4-D18*	this study
**KK1970**	*h+*	*nup60::Hygro ulp1-GFP:Kan cut11-mCherry:Hygro ade6-704 leu1-32 ura4-D18*	this study
**KK2287**	*h-*	*nup61::Hygro ulp1-GFP:Kan cut11-mCherry:Hygro ade6-704 leu1-32 ura4-D18*	this study
**KK2309**	*h+*	*nup124::Hygro ulp1-GFP:Kan cut11-mCherry:Hygro ade6-704 leu1-32 ura4-D18*	this study

**KK2071**	*h+*	*alm1::Hygro ulp1-GFP:Kan cut11-mCherry:Hygro ade6-704 leu1-32 ura4-D18*	this study
**KK2292**	*h-*	*npp106-GFP:Nat cut11-mCherry:Hygro ade6-704 leu1-32 ura4-D18*	this study
**KK1788**	*h+*	nup211-mAID-HA-Turg1:Kan ade6-704 leu1-32 ura4-D18	this study
**KK1790**	*h+*	nup211-mAID-HA-Turg1:Kan arg3::bleMX6-arg3+-padh1-OsTIR1F74A-Tadh1 ade6-704 leu1-32 ura4-D18	this study
**KK1780**	*h+*	ulp1-GFP:Kan nup211-mAID-HA-Turg1:Kan ade6-704 leu1-32 ura4-D18	this study
**KK1782**	*h-*	ulp1-GFP:Kan nup211-mAID-HA-Turg1:Kan arg3::bleMX6-arg3+-padh1-OsTIR1F74A-Tadh1 ade6-704 leu1-32 ura4-D18	this study
**KK2273**	*h-*	*SUMO-KallR (pmt3-KallR) nmt41:rtf1:sup35 ade6-704 leu1-32 t-ura4-SD20<ori (uraR)*	Kramarz et al., 2020
**KK2281**	*h-*	*nup60::Hygro SUMO-KallR (pmt3-KallR) nmt41:rtf1:sup35 ade6-704 leu1-32 t-ura4-SD20<ori (uraR)*	this study
**KK2403**	*h-*	*alm1::Hygro SUMO-KallR (pmt3-KallR) nmt41:rtf1:sup35 ade6-704 leu1-32 t-ura4-SD20<ori (uraR)*	this study
**KK2391**	*h+*	*rpn10::Hygro SUMO-KallR (pmt3-KallR) nmt41:rtf1:sup35 ade6-704 leu1-32 t-ura4-SD20<ori (uraR)*	this study
**KK1631**	*h-*	*nmt41:rtf1:sup35 ade6-704 leu1-32 t-Kan-lexBS:ura4-SD20<ori (uraR)*	Kramarz et al., 2020
**KK1635**	*h-*	*ulp1-lexA:Hygro nmt41:rtf1:sup35 ade6-704 leu1-32 t-Kan-lexBS:ura4-SD20<ori (uraR)*	Kramarz et al., 2020
**KK1639**	*h-*	*nup60::Hygro nmt41:rtf1:sup35 ade6-704 leu1-32 t-Kan-lexBS:ura4-SD20<ori (uraR)*	this study
**KK1642**	*h+*	*nup60::Hygro ulp1-lexA:Hygro nmt41:rtf1:sup35 ade6-704 leu1-32 t-Kan-lexBS:ura4-SD20<ori (uraR)*	this study
**KK1769**	*h-*	*alm1::Hygro nmt41:rtf1:sup35 ade6-704 leu1-32 t-Kan-lexBS:ura4-SD20<ori (uraR)*	this study
**KK1772**	*h-*	*alm1::Hygro ulp1-lexA:Hygro nmt41:rtf1:sup35 ade6-704 leu1-32 t-Kan-lexBS:ura4-SD20<ori (uraR)*	this study
**KK1192**	*h+*	*npp106-GFP:Nat arg3::mCherry-LacI nmt41:rtf1:sup35 ade6-704 leu1-32 t-LacO 7,9Kb:Kan:ura4::LexBS<ori (uraR)*	Kramarz et al., 2020
**KK1193**	*h-*	*ulp1-lexA:Hygro npp106-GFP:Nat arg3::mCherry-LacI nmt41:rtf1:sup35 ade6-704 leu1-32 t-LacO 7,9Kb:Kan:ura4::LexBS<ori (uraR)*	Kramarz et al., 2020
**KK1854**	*h-*	*nup60::Hygro npp106-GFP:Nat arg3::mCherry-LacI nmt41:rtf1:sup35 ade6-704 leu1-32 t-LacO 7,9Kb:Kan:ura4::LexBS<ori (uraR)*	this study
**KK1857**	*h-*	*nup60::Hygro ulp1-lexA:Hygro npp106-GFP:Nat arg3::mCherry-LacI nmt41:rtf1:sup35 ade6-704 leu1-32 t-LacO 7,9Kb:Kan:ura4::LexBS<ori (uraR)*	this study
**KK1931**	*h-*	*alm1::Hygro npp106-GFP:Nat arg3::mCherry-LacI nmt41:rtf1:sup35 ade6-704 leu1-32 t-LacO 7,9Kb:Kan:ura4::LexBS<ori (uraR)*	this study
**KK1929**	*h+*	*alm1::Hygro ulp1-lexA:Hygro npp106-GFP:Nat arg3::mCherry-LacI nmt41:rtf1:sup35 ade6-704 leu1-32 t-LacO 7,9Kb:Kan:ura4::LexBS<ori (uraR)*	this study
**KK1527**	*h-*	*rpn10::Hygro LoxP-cdc6-L591G-LoxM3 rtf1::Nat loxP-rnh201RED:KAN-loxM3 ChrII-8535:Rura RTS1::phleo ura4-D18 ade6-704 leu1-32*	this study

**KK1868**	*h-*	*rpn10::Hygro LoxP-cdc6-L591G-LoxM3 Nat:ADH1:rtf1 loxP-rnh201RED:KAN-loxM3 ChrII-8535:Rura RTS1::phleo ura4-D18 ade6-704 leu1-32*	this study
**KK1526**	*h-*	*rpn10::Hygro loxP-cdc20-M630F-loxM3 rtf1::Nat loxP-rnh201RED:KAN-loxM3 ChrII-8535:Rura RTS1::phleo ura4-D18 ade6-704 leu1-32*	this study
**KK1894**	*h-*	*rpn10::Hygro loxP-cdc20-M630F-loxM3 Nat:ADH1:rtf1 loxP-rnh201RED:KAN-loxM3 ChrII-8535:Rura RTS1::phleo ura4-D18 ade6-704 leu1-32*	this study

Supplementary Table 2: Primer list

**Name**	**Distance (bp) from the *RTS1*-RFB**	**sequence**
**L5F**	-110	AGGGCATTAAGGCTTATTTACAGA
**L5R**	-110	TCACGTTTAATTTCAAACATCCA
**L3F**	110	TTTAAATCAAATCTTCCATGCG
**L3R**	110	TGTACCCATGAGCAAACTGC
**L400F**	400	ATCTGACATGGCATTCCTCA
**L400R**	400	GATGCCAGACCGTAATGACA
**II50F**	Intergenic locus on ChrII	CACCGCAGTTCTACGTATCCT
**II50R**	Intergenic locus on ChrII	CGATGTAACGGTATGCGGTA
